# A new synthesis of Tyrian purple (6,6’-dibromoindigo) and its corresponding sulfonate salts

**DOI:** 10.3762/bjoc.22.10

**Published:** 2026-01-21

**Authors:** Holly Helmers, Mark Horton, Julie Concepcion, Jeffrey Bjorklund, Nicholas C Boaz

**Affiliations:** 1 Department of Chemistry, North Central College, 121 S. Loomis Street, Naperville, IL 60540, USA

**Keywords:** 6,6’-dibromoindigo, dye, sulfonation, synthesis, Tyrian purple

## Abstract

6,6’-Dibromoindigo is the major component of a historic pigment, famous since ancient times, known as Tyrian purple. In this work, we report a new strategy for the synthesis of 6,6’-dibromoindigo in four steps from *p*-bromotoluene in 14.5% overall yield. A key improvement in the reported synthesis is the oxidation of the benzylic methyl group of 4-bromo-2-nitrotoluene to 4-bromo-2-nitrobenzaldehyde, which is accomplished by benzylic bromination followed by a Kornblum oxidation. This gentle oxidation avoids the need for chromium trioxide-mediated or nitrone-based methods. While other published syntheses of 6,6’-dibromoindigo have resulted in higher overall yields, our approach offers the advantages of inexpensive starting reagents, operationally simple reactions, and minimal purification of intermediates. Moreover, this work reports the successful sulfonation of 6,6’-dibromoindigo, producing water-soluble derivatives of this historically relevant dye.

## Introduction

Tyrian purple, a reddish-purple dye, has been used for millennia to create vibrant, purple-colored textiles [1]. This coveted dye was produced in small quantities using large numbers of sea snails found in the eastern Mediterranean Sea. The relative rarity and expense of the dye resulted in purple clothing being associated with wealth and power [1–2]. More modern analysis indicates that Tyrian purple is composed of several different indigoids, with the predominant being 6,6’-dibromoindigo (**1**) [3–4].

**Scheme 1:**
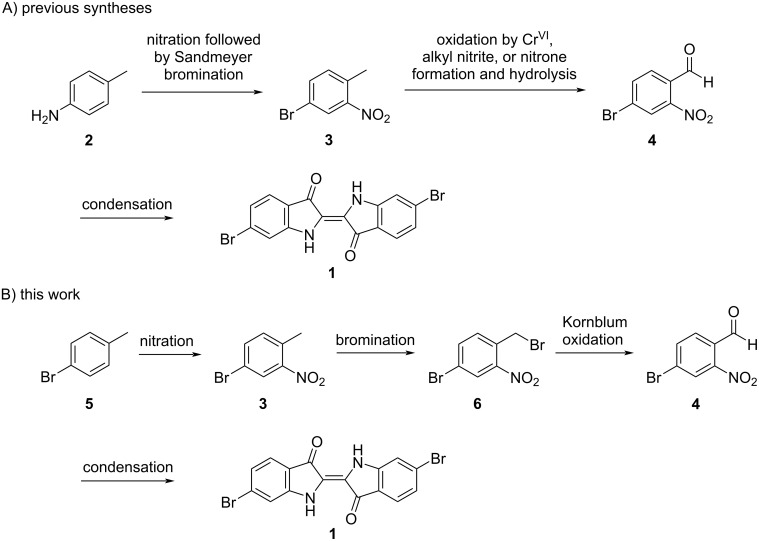
A) Generalized synthetic scheme for several previous syntheses of 6,6’-dibromoindigo. B) The synthetic scheme for 6,6’-dibromoindigo described in this work.

Since its discovery as the major component of Tyrian purple, several syntheses of **1** have been reported in the literature [5]. As shown in Scheme 1A, a common route to **1** uses 4-bromo-2-nitrotoluene (**3**) as a key intermediate [5]. We were interested in a safe and cost-efficient synthesis of the major component of Tyrian purple that is amenable to generating similar analogues, using **3** as a key intermediate. Furthermore, we were interested in producing a water-soluble derivative of **1**, much like indigo carmine was developed as a water-soluble derivative of indigo. Several syntheses of **3** have been reported, but the most common method involves the selective nitration of *p*-toluidine (**2**), followed by a Sandmeyer bromination [5–9]. While such chemistry is effective in smoothly generating **3** in good yield, this process requires the production of and use of a potentially explosive aryldiazonium intermediate [10]. Oxidation of the methyl group in **3** yields 4-bromo-2-nitrobenzaldehyde (**4**), which can then be directly converted into **1**. However, oxidation of the tolyl methyl group into an aldehyde has proved an ongoing challenge. Several syntheses have used a chromium(VI) oxidation to yield the desired product, but the toxic nature of this reagent and the expense of waste treatment raise issues [8–9,11–12]. Others have produced the desired aldehyde by formation and subsequent hydrolysis of a nitrone, a route which requires four distinct steps, making it less desirable [13–17].

While there have been several syntheses of **1** using **3** as a key intermediate, synthetic challenges remain. Specifically, the synthesis of **3** is challenging, often requiring Sandmeyer bromination to set the regiochemistry of the bromine [5–9]. Additionally, the oxidation of **3** to **4** often utilizes toxic chromium(VI) or a lengthy process [8–9,11–17]. In this work, we describe a more direct, chromium-free synthesis of **1** using inexpensive *p*-bromotoluene (**5**), as a starting material, which is also suitable for obtaining similar analogs. Moreover, we describe the sulfonation of **1**, which yields a water-soluble dye similar in properties to indigo carmine (5,5’-indigodisulfonic acid disodium salt).

## Results and Discussion

**Scheme 2:**
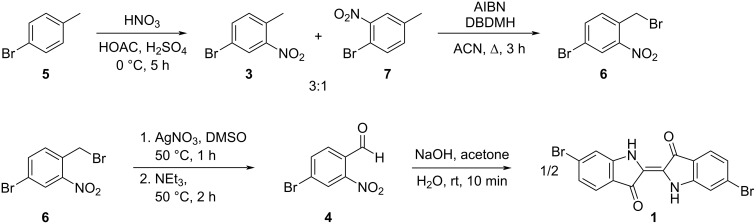
Synthetic scheme for the preparation of 6,6’-dibromoindigo from *p*-bromotoluene (**5**).

In pursuing a new synthesis of **1**, we sought to develop a shortened, chromium-free, synthetic approach leading to **1** via intermediates of **3** and **4**. Furthermore, we were interested in synthesizing a water-soluble derivative of **1** via sulfonation. In line with developing a shortened synthetic scheme, we chose *p*-bromotoluene (**5**), as a starting material, as nitration would yield **3** in a single step. Benzylic bromination followed by a Kornblum oxidation would then yield **4** in three steps from **5** without the use of a chromium(VI) oxidant or complex methods of oxidation. Condensation using established methods would then allow the synthesis of compound **1** in four steps overall, as shown in Scheme 2 [8,18].

**Scheme 3:**
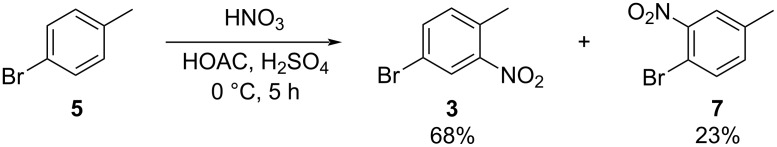
Nitration of *p*-bromotoluene (**5**) yields a mixture of regioisomers **3** and **7**.

To begin this synthesis, we needed to selectively nitrate *p*-bromotoluene (**5**), *ortho* to the methyl group, to yield **3**. Adapting the methodology of Keinan et al. [12], compound **5** was nitrated with fuming nitric acid in a mixture of glacial acetic acid and sulfuric acid. As shown in Scheme 3, this reaction yielded a mixture of **3** and 4-bromo-3-nitrotoluene (**7**) in a ≈3:1 ratio. Purification via column chromatography was successful in removing polynitrated side-products, but was unable to effectively separate the two regioisomers, and this mixture was carried on to the next step. The regioselectivity of nitration obtained in this reaction was better than the 5:4 ratio of **3** to **7** reported for a traditional nitration of *p*-bromotoluene [5,19]. Despite our best efforts, however, we were unable to reproduce the regioselectivity and 90% yield reported by Keinan et al. [12]. One possible explanation for this disparity in yield was the use of 90% fuming nitric acid in place of the reported 100% fuming nitric acid. At the time of this work, we were unable to purchase nitric acid of this grade from a commercial source. While **3** was produced as a mixture with its regioisomer **7**, this approach yielded the desired intermediate in a single step without the need for the high-energy diazonium intermediate used in other syntheses [6–9].

After having synthesized compound **3** via nitration of **5**, it was necessary to oxidize its methyl group to an aldehyde to yield 4-bromo-2-nitrobenzaldehyde (**4**). We sought to accomplish this transformation by first using a benzylic bromination of **3** to yield 4-bromo-2-nitrobenzyl bromide (**6**), followed by a Kornblum oxidation to produce **4**. This reaction sequence would mediate the oxidation of the tolyl methyl group of **3** to an aldehyde without the use of chromium(VI).

**Scheme 4:**
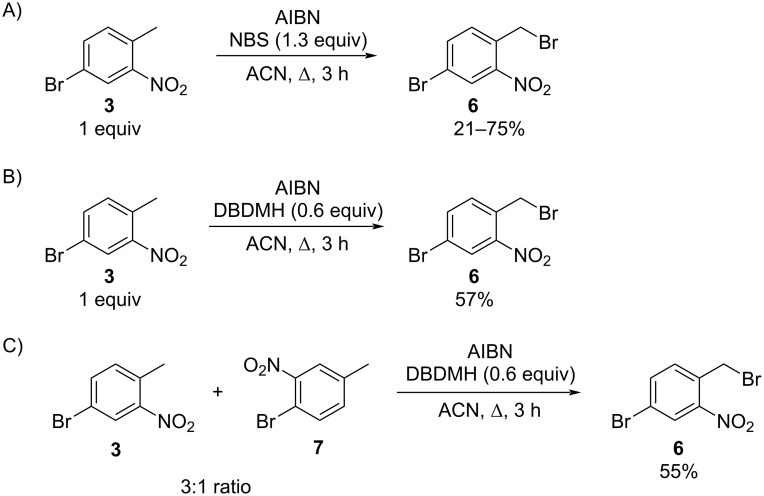
Benzylic bromination of 4-bromo-2-nitrotoluene (**3**).

Benzylic bromination of **3** was accomplished using a combination of a radical initiator and a bromine source. Initial optimization of the benzylic bromination reaction step of this strategy was done with commercially available **3**. As shown in Scheme 4A, reaction of **3** with *N*-bromosuccinimide (NBS) and azobisisobutyronitrile (AIBN) in acetonitrile, as previously described, led to benzylic bromination, but in inconsistent yields [20–21]. Use of 1,3-dibromo-5,5-dimethylhydantoin (DBDMH) as an alternative bromine source led to more consistent yields (57%) of the desired benzyl bromide (Scheme 4B). Systematic variation of the stoichiometric amount of DBDMH indicated that the yield of 4-bromo-2-nitrobenzylbromide (**6**) was optimized using 0.6 equiv or 1.2 equiv of available bromine (Scheme 4C). Similar yields, 55% relative to starting **3**, were obtained when the mixture of **3** and its regioisomer **7**, obtained from the previous step, was brominated using the optimized reaction conditions.

Compound **6** was purified and separated from any regioisomers by recrystallization from a mixture of hot toluene and hexanes. The efficacy of recrystallization was especially useful to our synthetic goal as it obviated the need to separate the mixture of regioisomers **3** and **7** from the nitration step, which were challenging to purify from each other. Product **6**, which was isolated as an off-white microcrystalline solid, was observed to be bench-stable for several months when stored in a sealed glass scintillation vial under ambient atmosphere at room temperature.

**Scheme 5:**
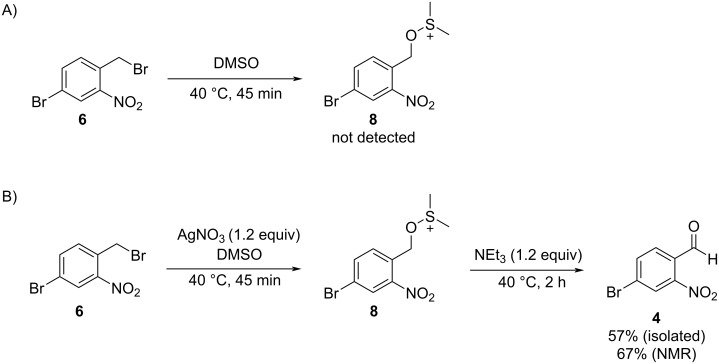
A) Treatment of 4-bromo-2-nitrobenzyl bromide (**6**) with DMSO did not yield the alkoxysulfonium ion intermediate **8**. B) Silver nitrate-mediated Kornblum oxidation yields 4-bromo-2-nitrobenzaldehyde (**4**) in fair yield. Please note that alkoxysulfonium ion **8** was not isolated.

Using the mild Kornblum oxidation, 4-bromo-2-nitrobenzyl bromide (**6**) was converted into the desired 4-bromo-2-nitrobenzaldehyde (**4**). As shown in Scheme 5A, dimethyl sulfoxide (DMSO) did not react with the benzyl bromide and instead only returned the starting material. The addition of 1.2 equivalents of silver nitrate promoted the substitution of the benzyl bromide with DMSO to yield an alkoxysulfonium ion **8** [22]. Following substitution, the addition of 1.2 equivalents of triethylamine mediated the formation of **4** in moderate yield, as shown in Scheme 5B (57% isolated, 67% based on NMR). The crude product was sufficiently pure (84 wt % by quantitative NMR) to carry on without purification, but further purification was possible by recrystallization from toluene and hexanes.

The two-step process of benzylic bromination and Kornblum oxidation of **3** to **4** was accomplished in 38% yield (37% if using the mixture of **3** and **7**). This oxidation is not the highest-yielding method for this transformation, but it represents a concise, operationally simple synthesis that does not require the use of chromium(VI) and, therefore, does not generate chromium waste that is costly to dispose of [23]. The literature reports that the oxidation of **3** to **4** can be performed using a high-valent chromium oxidation, followed by hydrolysis of the formed benzylidene diacetate with yields ranging from 18 to 60.5% yield relative to starting **3** [8–9,11–12]. Chromium-free oxidation of **3** to **4** has been reported via the condensation of **3** with an alkyl nitrate to yield an oxime. Subsequent hydrolysis of the oxime yields the desired aldehyde **4**, but only in low yield [6–7]. Finally, Kröhnke et al. reported a 4-step conversion of **3** to **4** that involves formation and hydrolysis of a nitrone to yield the desired aldehyde in 55% yield, but multiple steps make this pathway cumbersome [13–17,20]. Therefore, the route of benzylic oxidation that we report provides a reaction pathway most consistent with our goals.

**Scheme 6:**
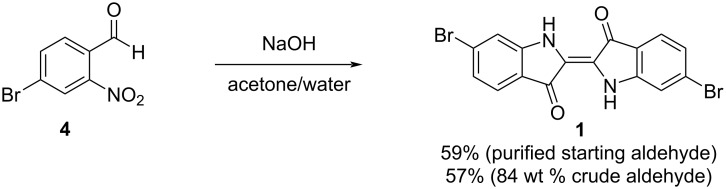
Condensation of 4-bromo-2-nitrobenzaldehyde (**4**) to yield 6,6’-dibromoindigo (**1**).

Condensation of 4-bromo-2-nitrobenzaldehyde (**4**) to 6,6’-dibromoindigo (**1**) was accomplished via the Baeyer–Drewson process initially developed for the synthesis of indigo [8,18,24]. As shown in Scheme 6, treatment of **4** with sodium hydroxide in aqueous acetone yields **1** as a dark purple powder in 59% isolated yield. It was also possible to use crude **4** obtained in the previous step (84% by wt) without purification to yield **1** in 56% isolated yield. This yield is similar to others reported for the condensation of **4** to **1** using the Baeyer–Drewson process [7–9].

**Scheme 7:**
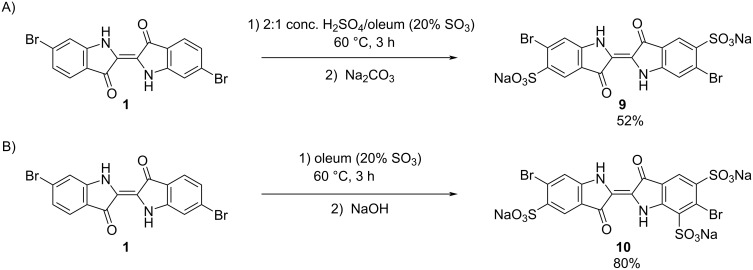
A) Disulfonation of 6,6’-dibromoindigo (**1**), to yield 6,6’-dibromo-5,5’-indigodisulfonic acid disodium salt (**9**). B) Trisulfonation of 6,6’-dibromoindigo (**1**), to yield 6,6’-dibromo-5,5’,7-indigotrisulfonic acid trisodium salt (**10**).

Analogous to the sulfonation of indigo to synthesize the water-soluble dye indigo carmine, compound **1** was sulfonated to increase its water solubility. Thus, the desired 6,6’-dibromo-5,5’-indigodisulfonic acid disodium salt (**9**) was obtained by heating **1** in a 2:1 mixture of oleum (20% free SO_3_) and concentrated sulfuric acid (Scheme 7A). The trisulfonated 6,6’-dibromo-5,5’,7-indigotrisulfonic acid trisodium salt (**10**) was produced by heating **1** in oleum (20% free SO_3_), as shown in Scheme 7B. Although monosulfonation of indigo was reported by Sullivan and co-workers, an analogous reaction was not able to be achieved for **1** [25]. The sulfonation of **1** appears to be more challenging than the corresponding sulfonation of indigo, which requires less forcing conditions [25]. This suggests that the bromine substituents deactivate the reactivity of the aromatic ring. As was reported for sulfonated indigo derivatives, an increasing number of sulfonic acid groups results in greater solubility of the 6,6’-dibromoindigo derivative (e.g., **10** has greater solubility in water than **9**) [25].

**Figure 1:**
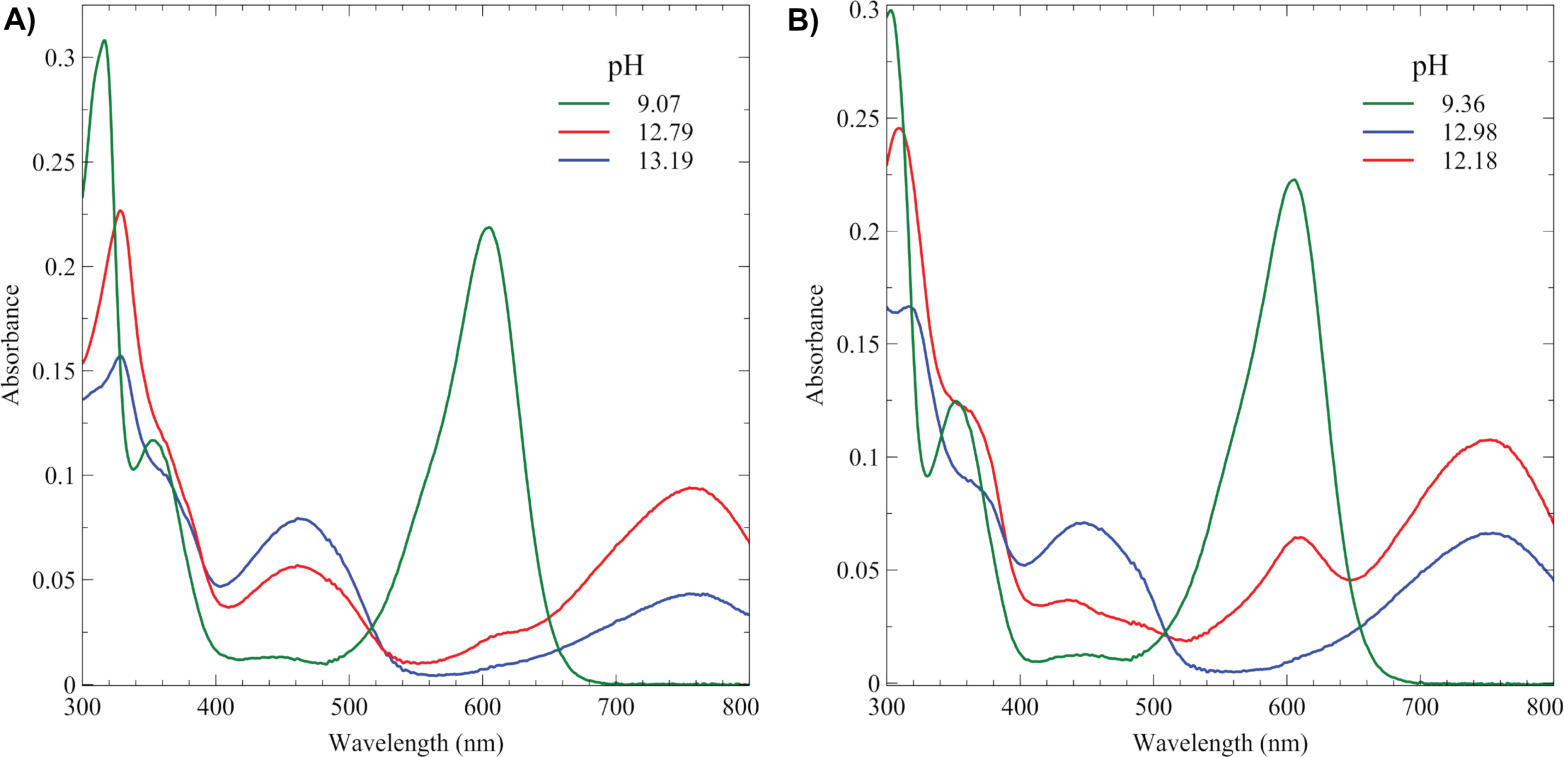
A) UV–vis spectra of 6,6’-dibromo-5,5’,7-indigotrisulfonic acid trisodium salt (**10**) (10 μM) in aqueous solution at different pH values. B) UV–vis spectra of 6,6’-dibromo-5,5’-indigodisulfonic acid disodium salt (**9**) (10 μM) in aqueous solution at different pH values.

When dissolved in aqueous solution, both **9** and **10** yield a bright blue solution. As shown in Figure 1A, salt **10** displays a λ_max_ at 605 nm at neutral to slightly alkaline conditions with additional absorptions at 354 nm and 315 nm. As the pH of the solution increases from neutral values to 12.3, there is a transition to a new λ_max_ at 756 nm with additional absorptions at 461 nm and 326 nm. Isosbestic points at 648 nm and 508 nm suggest only two species are in solution. Plotting absorbance versus pH reveals a p*K*_a_ value of 11.3 ± 0.05 (Supporting Information File 1, Figure S25). Additional increases in pH lead to a decrease in the absorbance at 755 nm and a growing absorbance at 461 nm indicating a second deprotonation. Examining the absorbance at 755 nm versus pH yields a second p*K*_a_ value at 13.0 ± 0.11 (Supporting Information File 1, Figure S26). As shown in Figure 1B, the corresponding disulfonate of 6,6’-dibromoindigo, **9**, shows similar UV–vis spectra to compound **10**. Specifically, **9** shows a λ_max_ at 606 nm and absorptions at 354 nm and 304 nm. As the pH of the solution increases from neutral values to 12.18, there is a transition to a new λ_max_ at 749 nm with additional absorptions at 435 nm and 309 nm. Isosbestic points at 648 nm and 515 nm indicate that between neutral pH and 12.18 only two species are in solution. Plotting absorbance versus pH yields a p*K*_a_ value of 11.9 ± 0.05 (Supporting Information File 1, Figure S17). Above a pH of 12.18, a peak at 448 nm grows while the peak at 749 nm decreases (Supporting Information File 1, Figure S18). The changes in the visible spectra of **9** above a pH of 12.18 no longer create isosbestic points, indicating that there are likely more than two unique species in solution. We believe that this indicates the presence of a second p*K*_a_ value, as was the case for **10**. Because of how close the second p*K*_a_ value is to the first p*K*_a_ value, we were unable to determine the exact value for this second deprotonation, but we estimate it to be above 12.5. Finally, analogously with 5,5’-indigodisulfonic acid (indigo carmine), both **9** and **10** can be readily reduced to their leuco forms using sodium dithionite, yielding a clear solution (Supporting Information File 1, Figures S19 and S27) [26].

The UV–vis spectra of both **9** and **10** are similar to those of 5,5’-indigodisulfonic acid disodium salt (indigo carmine) and 5,5’,7-indigotrisulfonic acid trisodium salt, as shown in Table 1 [27–28]. When compared to the absorption maximum of 6,6’-dibromoindigo (**1**), di- and trisulfonation causes a red shift by 16 and 15 nm with a moderate increase in molar absorptivity, respectively, for compounds **9** and **10** [29]. This means that to the human eye, di- and trisulfonated derivatives of **1** appear blue, not purple. A smaller shift in λ_max_ is observed when indigo is sulfonated, allowing for its blue color to be retained in its sulfonated derivatives [27–29]. As shown in Table 1, the p*K*_a_ values of **9** and **10** each have first p*K*_a_ values about 0.4 p*K* units below their non-brominated derivatives, indicating the impact of the electron-withdrawing halogen substituents [30].

**Table 1:** p*K*_a_ Values and λ_max_ values for sulfonated indigo and 6,6’-dibromoindigo species.

Species	p*K*_a1_	p*K*_a2_	λ_max_ (nm), ε (M^−1^cm^−1^)

indigo			605,16580^a^ [29]
5,5’-indigodisulfonic acid disodium salt	12.3 ± 0.2 [30]	12.8 ± 0.2 [30]	608–613, [31] 610, 20000 [27] 615, 20200 [32]
5,5’,7-indigotrisulfonic acid trisodium salt	11.7 ± 0.2 [30]	12.8 ± 0.2 [30]	600, 20000 [27]
6,6’-dibromoindigo (**1**)			590,17300^a^ [29]
6,6’-dibromo-5,5’-indigodisulfonic acid disodium salt (**9**)	11.9 ± 0.05	>12.5	606, 21500
6,6’-dibromo-5,5’,7-indigotrisulfonic acid trisodium salt (**10**)	11.3 ± 0.05	13.0 ± 0.11	605, 25000

^a^Determined in tetrachloroethane.

## Conclusion

Overall, the synthesis of 6,6’-dibromoindigo (**1**) was accomplished in 14.5% yield over 4 steps starting from *p*-bromotoluene (**5**). To the best of our knowledge, this work is the first to report the synthesis of compound **1** using **5** as a starting material, which is readily amenable to the synthesis of analogs. Additionally, this work performs the oxidation of the methyl group in 4-bromo-2-nitrotoluene to an aldehyde using a strategy of radical bromination followed by a Kornblum oxidation to give 4-bromo-2-nitrobenzaldehyde. This transformation eliminates the need for a chromium trioxide-mediated oxidation and the expenses of associated waste disposal or the lengthy conversion involving the formation and hydrolysis of a nitrone [7–9,13–17]. When compared to other published syntheses of **1**, the overall yield is reasonable, although others have reported strategies with higher yields. For example, Imming and co-workers reported a 10% yield over 5 steps from *p*-toluidine, Wolk and Frimer reported a 25% yield over 5 steps from *p*-dibromobenzene, while Voß and Gerlach reported a yield of 61% over 3 steps from 1,4-dibromo-2-nitrobenzene, although specialized techniques were required [8,33–34]. We believe that the reported synthetic scheme is valuable because it can produce 6,6’-dibromoindigo from an inexpensive starting material using operationally simple reactions without the need for extensive purification of intermediates. Additionally, this work shows that it is possible to sulfonate compound **1**, thereby creating water-soluble derivatives of this ancient dye.

## Supporting Information

File 1 Experimental procedures, characterization data and copies of spectra.

## Data Availability

Data generated and analyzed during this study is available from the corresponding author upon reasonable request.
