# Supplementary material for: A new synthesis of Tyrian purple (6,6’-dibromoindigo) and its corresponding sulfonate salts
Source: Beilstein J Org Chem. 2026 Jan 21;22:167–74. doi: 10.3762/bjoc.22.10 (PMC12834052; doi:10.3762/bjoc.22.10)
Supplement: File 1 — Experimental procedures, characterization data and copies of spectra. [file Beilstein_J_Org_Chem-22-167-s001.pdf]

BEILSTEIN JOURNAL OF ORGANIC CHEMISTRY

Supporting Information

for

A new synthesis of Tyrian purple (6,6'-dibromoindigo) and its corresponding sulfonate salts

Holly Helmers, Mark Horton, Julie Concepcion, Jeffrey Bjorklund and Nicholas C. Boaz

Beilstein J. Org. Chem. **2026,** *22,* 167–174. doi:10.3762/bjoc.22.10

Experimental procedures, characterization data and copies of spectra

License and Terms: This is a supporting information file under the terms of the Creative Commons Attribution License (https://creativecommons.org/licenses/by/4.0). Please note that the reuse, redistribution and reproduction in particular requires that the author(s) and source are credited and that individual graphics may be subject to special legal provisions.
The license is subject to the *Beilstein Journal of Organic Chemistry* terms and conditions: (https://www.beilstein-journals.org/bjoc/terms)

Table of contents

General experimental details........S2

Synthetic details........S2

Spectral data of synthesized compounds........S6

References........S27

Caution: Many of the reagents and conditions outlined in this paper are potentially dangerous. Please ensure that proper precautions are taken and literature is consulted when working with oxidizers and strong acids.

General experimental details:
Unless otherwise indicated, all reagents were used as received without further purification. Denatured ethanol and *p*-bromotoluene (98%) were obtained from Beantown Chemical. Sulfuric acid (ACS reagent, 95–98%), fuming sulfuric acid (ACS reagent, 20% free SO_3), 1,3-dibromo-5,5-dimethylhydantoin (98%), dimethyl sulfoxide (>99.5%), and triethylamine (>99.5%) were obtained from Sigma Aldrich. 4-Bromo-2-nitrotoluene (>98%) was obtained from TCI America. Sodium hydroxide and sodium acetate (ACS reagent) were purchased from Research Products International. Silver nitrate (>99.7%) was obtained from Alpha Division. Glacial acetic acid (ACS reagent) was obtained from GFS Chemicals. Dichloromethane (ACS reagent), acetonitrile (ACS reagent), hexanes (ACS reagent), ethyl acetate (ACS reagent), and acetone (ACS reagent) were obtained from Pharmco.

NMR analyses were performed using a Bruker Avance 400 MHz spectrometer equipped with a multinuclear probe. GC–MS analyses were performed using an Agilent 8860 gas chromatograph paired with an Agilent 5977C mass spectral detector. FT-IR spectra were obtained using a Perkin Elmer Spectrum 100 equipped with an ATR stage. UV–vis spectra were obtained using an Agilent Cary double-beam spectrophotometer.

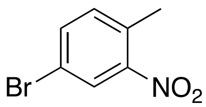

4-Bromo-2-nitrotoluene (3): The nitration of *p*-bromotoluene was performed using the method of Keinan et al. [1]. Briefly, a 100 mL flask equipped with a stirring bar and an addition funnel was charged with *p*-bromotoluene (10.686 g, 62.5 mmol, 1 equiv), glacial acetic acid (9.1 mL), and concentrated sulfuric acid (8.8 mL). The reaction mixture was allowed to stir at room temperature until homogeneous. The reaction flask was then cooled to 0 °C, and a mixture of nitric acid (3.6 mL, 90%) and sulfuric acid (8.8 mL) was added dropwise over 5 hours. The reaction was allowed to warm to room temperature and stirred overnight. The mixture was then poured over ground ice (100 g) and extracted with methylene chloride (2 × 50 mL). The organic layer was then washed with saturated aqueous sodium carbonate (3 × 20 mL), dried over anhydrous sodium sulfate, and concentrated in vacuo. The crude mixture was purified via flash chromatography using silica gel, eluting with ethyl acetate/hexanes. The title compound, a yellow oil, was obtained as a mixture with its regioisomer, 4-bromo-3-nitrotoluene (12.27 g, 75:25 4-bromo-2-nitrotoluene to 4-bromo-3-nitrotoluene, 68%). The product was characterized via GC–MS, and the retention time and mass spectrum matched that of an authentic standard.

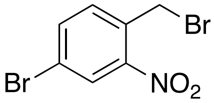

4-Bromo-2-nitrobenzyl bromide (6): A 250 mL round-bottomed flask equipped with a stirring bar and a water-cooled reflux condenser was charged with 4-bromo-2-nitrotoluene (10.8 g, 50 mmol, 1 equiv), 1,3-dibromo-5,5-dimethylhydantoin (8.57 g, 30 mmol, 0.6 equiv), and azobisisobutyronitrile (498 mg, 3 mmol, 6 mol %). Acetonitrile (60 mL) was added and the mixture was

heated at vigorous reflux for 3 hours. The mixture was then cooled, and the solvent was removed in vacuo. The solid mixture was then redissolved in methylene chloride (50 mL) and washed with concentrated aqueous sodium metabisulfite (3 × 20 mL). The combined organic washings were then dried over anhydrous sodium sulfate and concentrated in vacuo. The crude product was then dissolved in 5 mL of hot toluene. Hot hexanes (15 mL) were then added to the solution, and the product was allowed to cool to room temperature slowly. After reaching room temperature, the solution was stored overnight at 4 °C, allowing the product to crystallize as off-white needles. The crystalline product was isolated via filtration and washed with cold hexanes (3 × 5 mL). The title compound was yielded as a light brown to off-white solid (8.45 g, 57% yield). The product was characterized by ^{1}H NMR, ^{13}C NMR, IR, and EIMS and matched spectral data previously reported [2].

4-Bromo-2-nitrobenzylbromide may also be synthesized via the described bromination procedure using the crude 4-bromo-2-nitrotoluene (3:1mixture of 4-bromo-2-nitrotoluene and 4-bromo-3-nitrotoluene) obtained by nitration of 4-bromotoluene described above. Starting with 10.8 g of crude 4-bromo-2-nitrotoluene (3:1 ratio of 4-bromo-2-nitrotoluene and 4-bromo-3-nitrotoluene), the above bromination procedure yielded 4-bromo-2-nitrobenzyl bromide as a light brown to off white solid (6.0 g, 55% yield relative to starting 4-bromo-2-nitrotoluene).

^{1}H NMR ($CDCl_3$, 400 MHz, δ) 8.21 (d, *J* = 2.08 Hz, 1 H), 7.77 (dd, *J* = 8.38, 2.02 Hz, 1 H), 7.48 (d, *J* = 8.20 Hz, 1 H), 4.80 (s, 2H) ^{13}C NMR ($CDCl_3$, 100 MHz, δ) 148.2, 136.8, 133.8, 131.8, 128.5, 122.9, 28.0. IR (ATR) v 3085, 1523, 1479, 1434, 1342, 1223 cm^{-1}. EI MS $C_7H_5Br_2NO_2$ calculated 294.866 m/z observed 294.9 m/z.

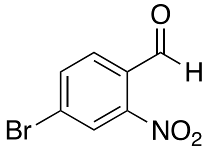

4-Bromo-2-nitrobenzaldehyde (4): A 25 mL round-bottomed flask equipped with a stirring bar was charged with 4-bromo-2-nitrobenzylbromide (5.90 g, 20 mmol, 1 equiv) and DMSO (20 mL). After dissolution of the 4-bromo-2-nitrobenzylbromide, silver(I) nitrate (5.08 g, 30 mmol, 1.5 equiv) was added. The reaction was stirred at 40 °C for 45 minutes. During this time, a fine green precipitate formed. Triethylamine (4 mL, 30 mmol, 1.5 equiv) was added, and the reaction mixture was allowed to stir at 40 °C for 2 hours. The reaction was diluted into 50 mL of methylene chloride and filtered to remove the silver bromide precipitate. The reaction was then washed with 1 M H_2SO_4 (4 × 50 mL). The combined organic washings were then dried over sodium sulfate and concentrated in vacuo. The crude product was purified via recrystallization from a mixture of toluene and hexanes. The title compound was obtained as a white solid (2.61 g, 57% yield). The product was characterized by ^{1}H NMR, ^{13}C NMR, IR, and EIMS and matched spectral data previously reported [3].

^{1}H NMR ($CDCl_3$, 400 MHz, δ) 10.41 (d, *J* = 0.49 Hz, 1H), 8.29 (d, *J* = 1.83 Hz, 1H), 7.96 (ddd, *J* = 8.28, 1.86, 0.70 Hz), 7.87 (d, *J* = 8.19 Hz, 1H). ^{13}C NMR ($CDCl_3$, 100 MHz, δ) 187.0, 149.9 137.2, 131.0, 129.7, 128.2, 127.7 IR (ATR) v 3092, 2912, 1686, 1590, 1556, 1523, 1342 cm^{-1}. EI MS $C_7H_5Br_2NO_2$ calculated 228.937 m/z observed 229.0 m/z.

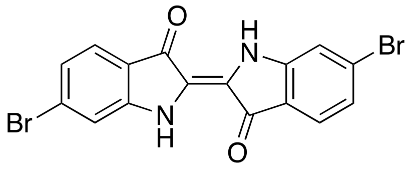

6,6'-Dibromoindigo (1): The synthesis of 6,6'-dibromoindigo was performed using the procedure detailed by Winum et al. [4]. Briefly, a 200 mL round-bottomed flask equipped with a magnetic stirring bar was charged with 4-bromo-2-nitrobenzaldehyde (1 g, 4.3 mmol, 1 equiv), acetone (13 mL), and water (36 mL). Sodium hydroxide (2 M, 3.3 mL) was then added dropwise to the vigorously stirred solution. The solution was allowed to stir at room temperature for 10 minutes, during which time a deep purple color developed. The solution was then filtered using a 60 mL tared, fine-porosity sintered glass filter and washed with deionized water until the filtrate was clear, and then washed with 20 mL of ethanol. The product was isolated as a purple powder (0.547 g, 59%). The title compound was characterized by IR spectroscopy and matched the spectral data previously reported.[5]

6,6'-Dibromoindigo may also be produced via the described method using the impure 4-bromo-2-nitrobenzaldehyde (84 wt %) produced above. Starting with 1.176 g of crude 4-bromo-2-nitrobenzaldehyde (84 wt %), the above cyclization procedure yielded 6,6'-dibromoindigo as a dark purple solid (0.505 g, 56%).

IR (ATR) v 3383, 3081, 1632, 1611, 1576, 1436, 1155 cm^{-1}.

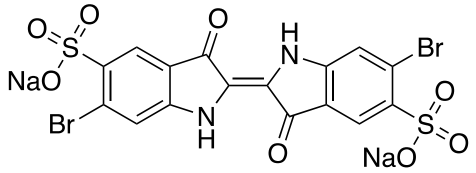

6,6'-Dibromo-5,5'-indigodisulfonic acid sodium salt (9): The sulfonation of 6,6'-dibromoindigo was adapted from Sullivan et al. [6]. A 20 mL scintillation vial equipped with a stirring bar was charged with 6,6'-dibromoindigo (420 mg, 1.0 mmol), concentrated sulfuric acid (4 mL), and oleum (2 mL, 20% free SO_3). The reaction mixture was then stirred at 60 °C for three hours. The solution, after cooling to room temperature, was diluted into 100 mL of cold deionized water. The resultant solution was then heated to a boil and filtered hot. After cooling to room temperature, the solution was neutralized slowly with sodium carbonate, yielding a blue precipitate. The precipitate was isolated via filtration using a fine-porosity fritted glass funnel. The crude blue precipitate was purified by recrystallization from a minimum amount of water to yield a blue-purple powder (323 mg, 52% yield).

^{1}H NMR (d_6DMSO, 400 MHz, 50°C, δ) 10.61 (s (broad), 2H), 8.12 (s, 2H), 7.56 (s, 2H). ^{13}C NMR (d_6DMSO, 100 MHz, 50°C δ) 187.44, 152.87, 140.16, 128.42, 124.13, 123.94, 118.59, 117.57. IR (ATR) v 3402, 1644, 1599, 1445, 1154, 1103, 1034 cm^{-1}. HR ESI-MS expected $C_{16}H_9Br_2N_2O_8S_2$ $[M-2Na^{+}+3H^{+}]^{+}$ 578.8167 m/z, observed 578.8162 m/z. UV-VIS (λ, nm; ε, $M^{-1}cm^{-1}$, pH 7.0, 100 mM phosphate acetate buffer): 606 (21500), 354 (12300), 304 (29500)

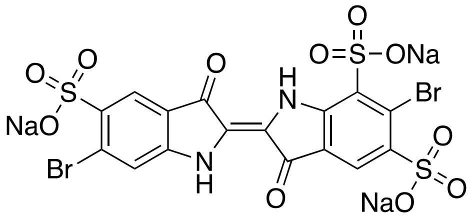

6,6'-Dibromo-5,5',7-indigotrisulfonic acid trisodium salt (10): The sulfonation of 6,6'-dibromoindigo was adapted from Sullivan et al. [6]. Briefly, a 25 mL round-bottomed flask equipped with a stirring bar and a rubber septum was charged with 6,6'-dibromoindigo (480 mg, 1.14 mmol, 1 equiv) and oleum (2 mL, 20% free SO_3). The reaction mixture was gently heated to 60 °C and stirred for 3 hours. The reaction mixture was then cooled to 0 °C using an ice bath and quenched via the careful addition of 25 mL of cold deionized water. The mixture was allowed to stir for 10 minutes, and concentrated sodium hydroxide was added until the pH of the solution reached a value >4.0. The solution was cooled to 0 °C overnight and filtered through a tared 60 mL fine porosity sintered glass funnel. The precipitate was resuspended in the glass funnel with saturated sodium acetate (50 mL) and filtered. The precipitate was then resuspended in the glass funnel with ethanol (50 mL, anhydrous) and filtered. The title product was obtained as a deep blue powder (659 mg, 80%).

^{1}H NMR (D_2O, 100 mM NaH_2PO_4, 400 MHz, δ) 8.18 (s, 1H), 8.03 (s, 1H), 7.30 (s, 1H) ^{13}C NMR (D_2O, 100 mM NaH_2PO_4,100 MHz, δ) 187.86, 186.19, 152.93, 148.52, 136.19, 134.60, 128.29, 127.73, 127.22, 127.15, 125.05, 121.96, 121.07, 119.04, 118.95, 117.15 IR (ATR) v 3371, 1635, 1597, 1430, 1184, 1041 cm^{-1}. HR ESI-MS expected $C_{16}H_9Br_2N_2O_{11}S_3$ [M-3Na$^+$+4H$^+$]$^+$ 658.7735 m/z, observed 658.7730 m/z UV-VIS (λ, nm; ε, M^{-1}cm^{-1}, pH 7.0 100 mM phosphate acetate buffer): 605 (25000), 354 (13500), 315 (34900).

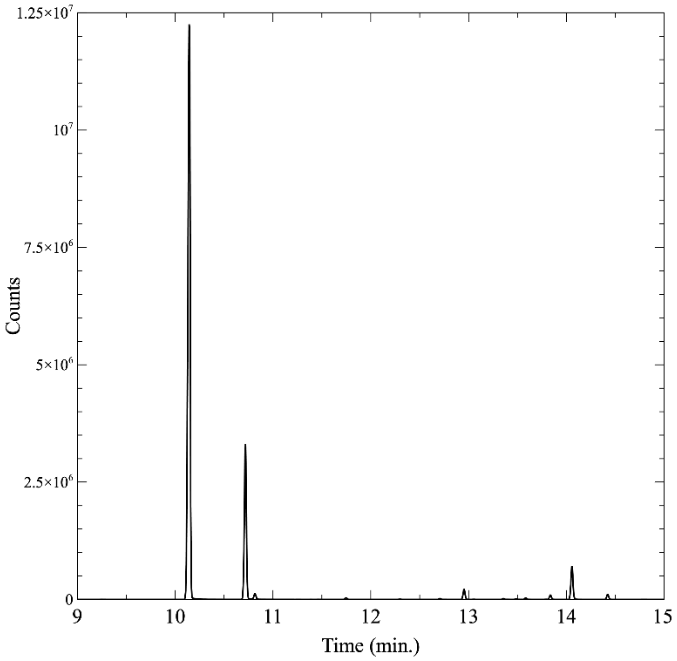

Figure S1: Gas chromatogram of the crude mixture obtained by nitration of *p*-bromotoluene (**5**). The desired 4-bromo-2-nitrotoluene (**3**) eluted at 10.1 minutes while the undesired 4-bromo-3-nitrotoluene (**7**) eluted at 10.7 minutes. Small amounts of polynitrated products eluted between 12.9 and 14.5 minutes.

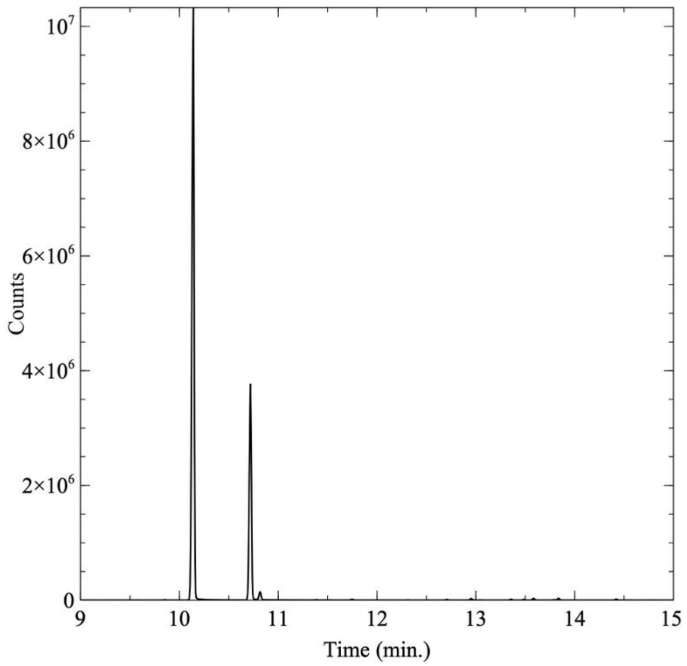

Figure S2: Gas chromatogram of the partially purified 4-bromo-2-nitrotoluene (**3**). The desired 4-bromo-2-nitrotoluene eluted at 10.1 minutes, while the undesired 4-bromo-3-nitrotoluene (**7**), eluted at 10.7 minutes. Please note that column chromatography effectively removed polynitrated impurities that elute between 12.9 and 14.5 minutes.

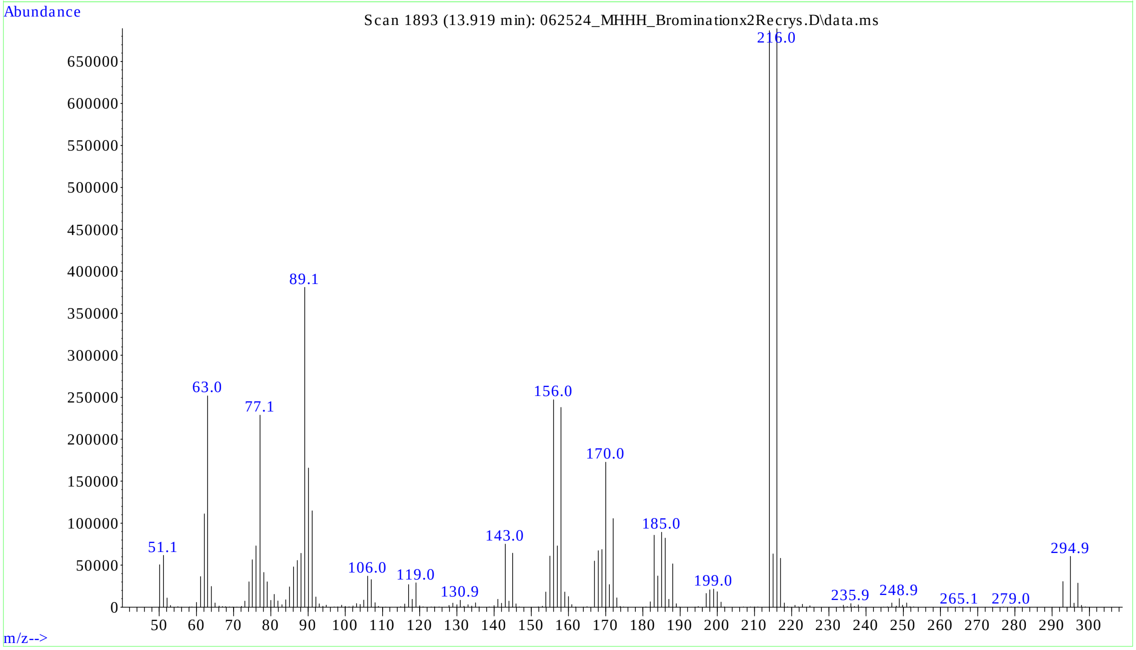

Figure S3: EI-Mass spectrum of 4-bromo-2-nitrobenzyl bromide (**6**).

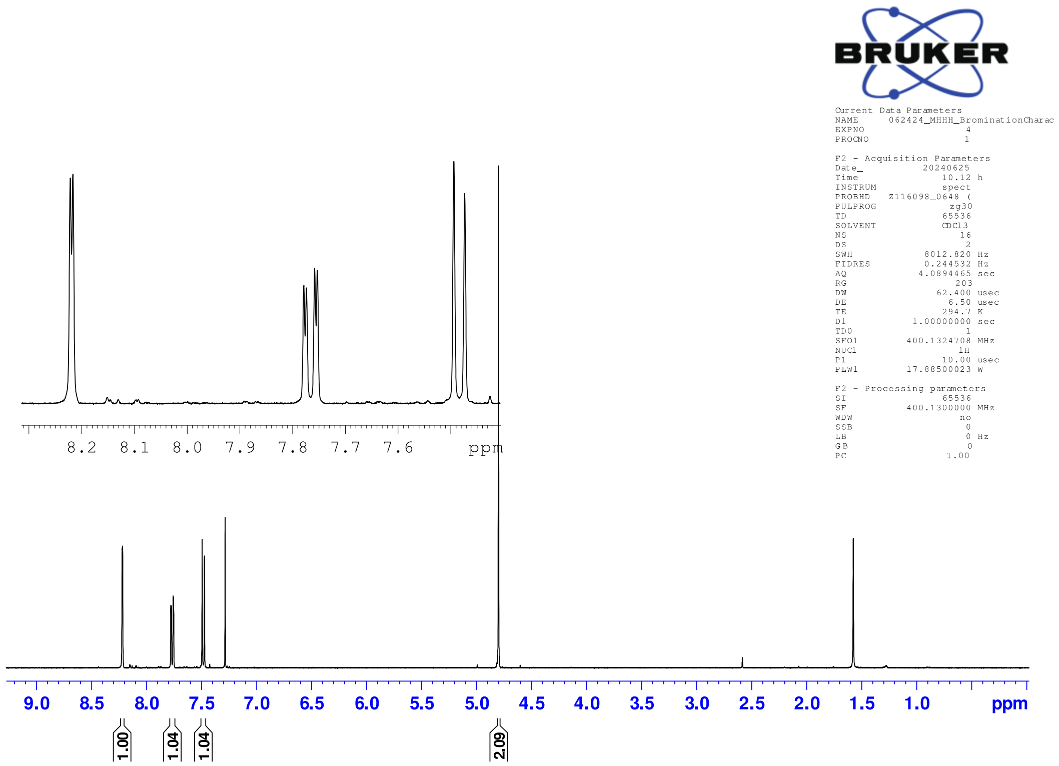

Figure S4: ^{1}H NMR of 4-bromo-2-nitrobenzyl bromide (**6**).

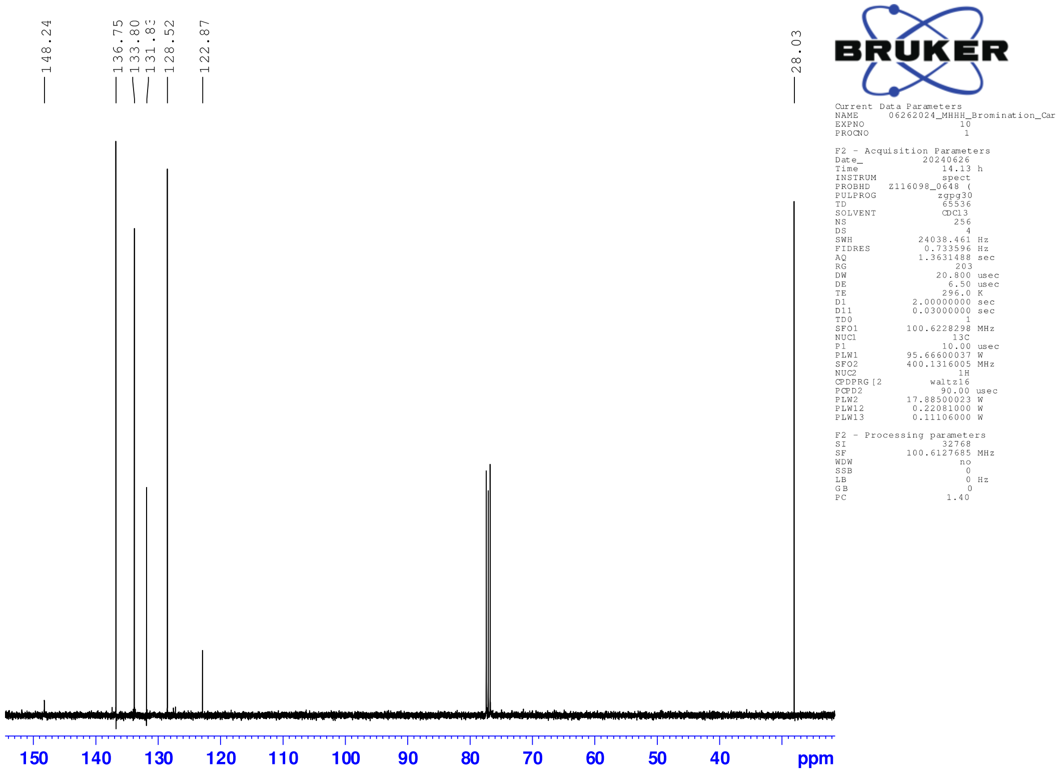

Figure S5: ^{13}C NMR of 4-bromo-2-nitrobenzyl bromide (**6**).

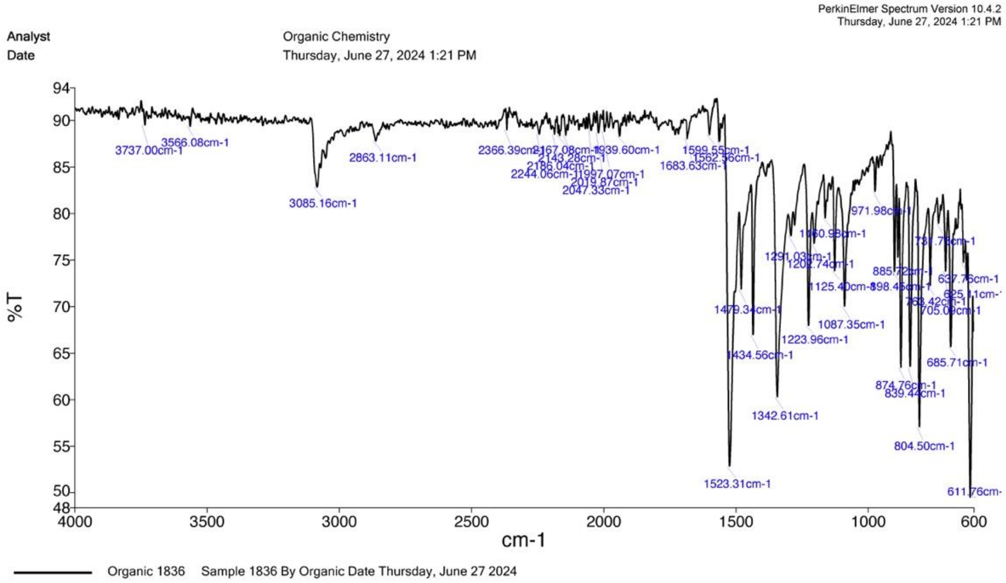

Figure S6: FT-IR spectrum of 4-bromo-2-nitrobenzyl bromide (**6**).

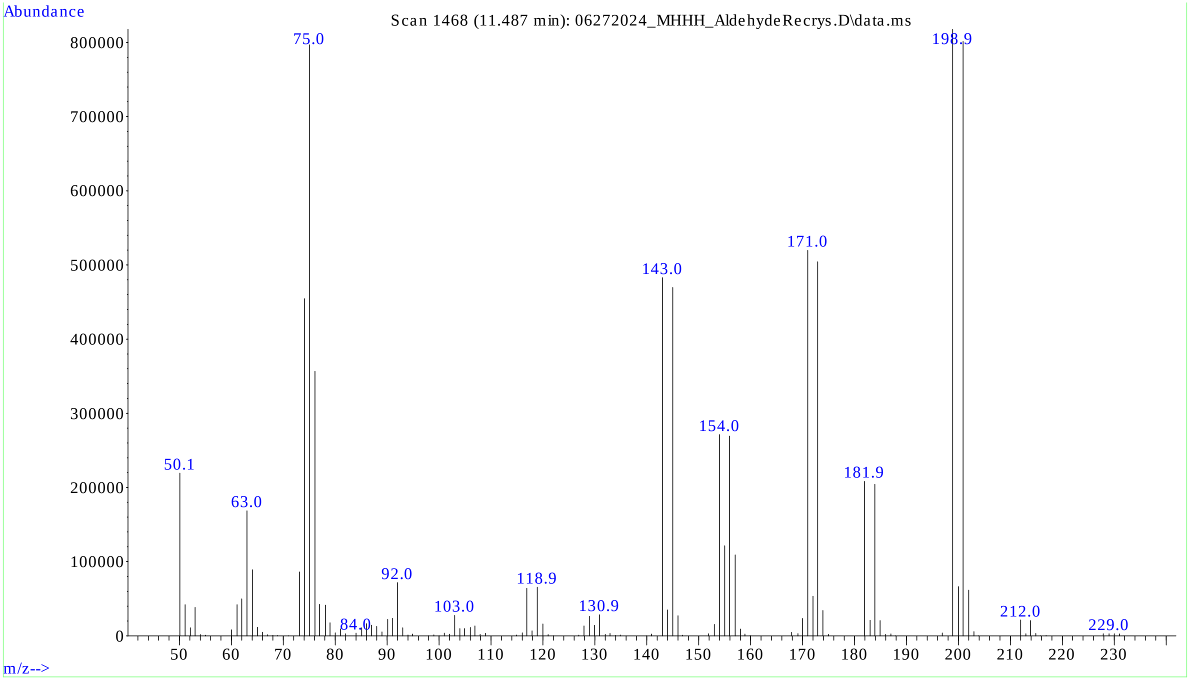

Figure S7: EIMS of 4-bromo-2-nitrobenzaldehyde (**4**).

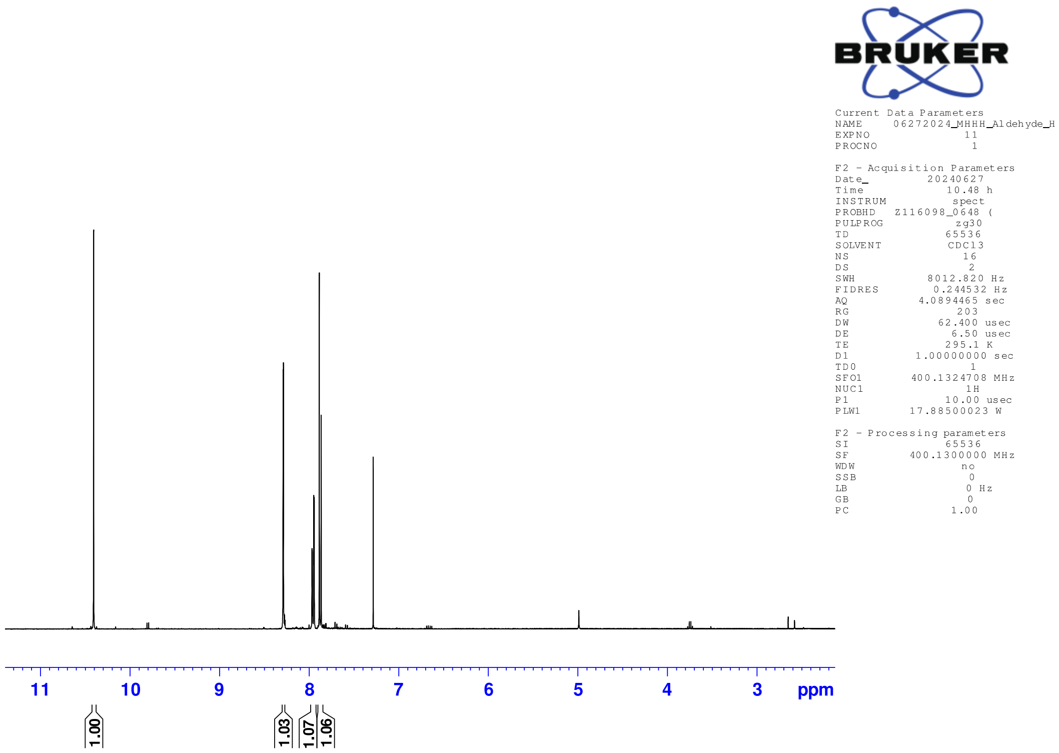

Figure S8: ^{1}H NMR of 4-bromo-2-nitrobenzaldehyde (**4**).

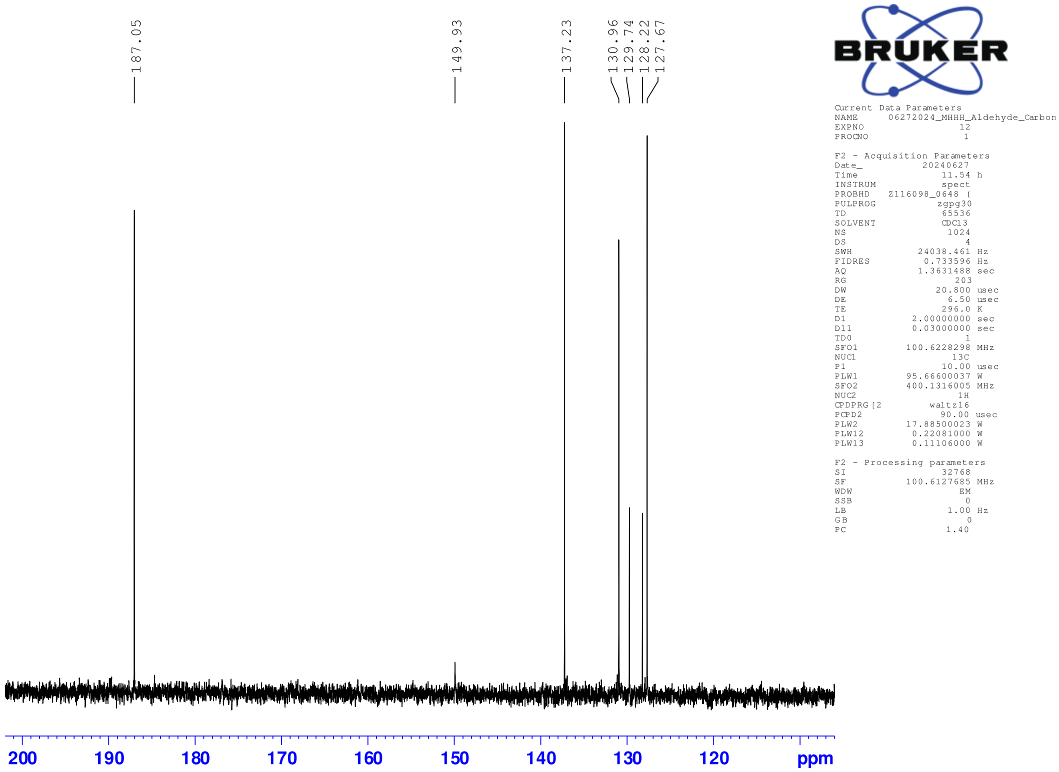

Figure S9: ^{13}C NMR of 4-bromo-2-nitrobenzaldehyde (**4**).

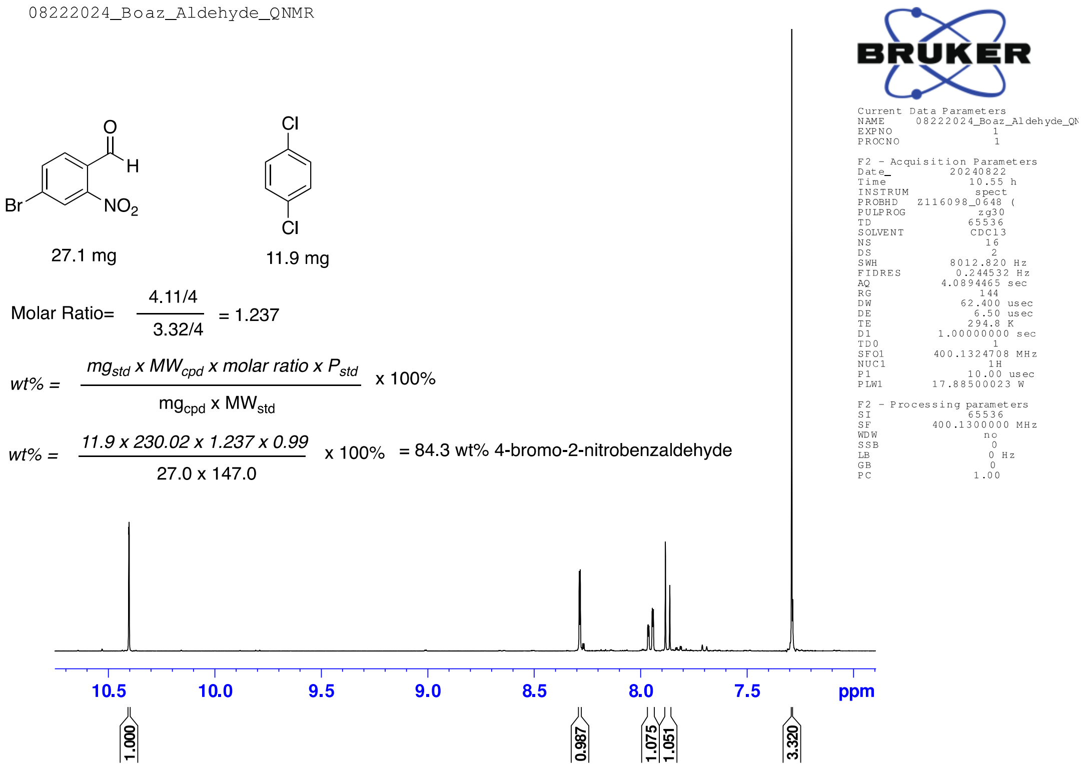

Figure S10: Quantitative ^{1}H NMR analysis of crude 4-bromo-2-nitrobenzaldehyde (**4**).

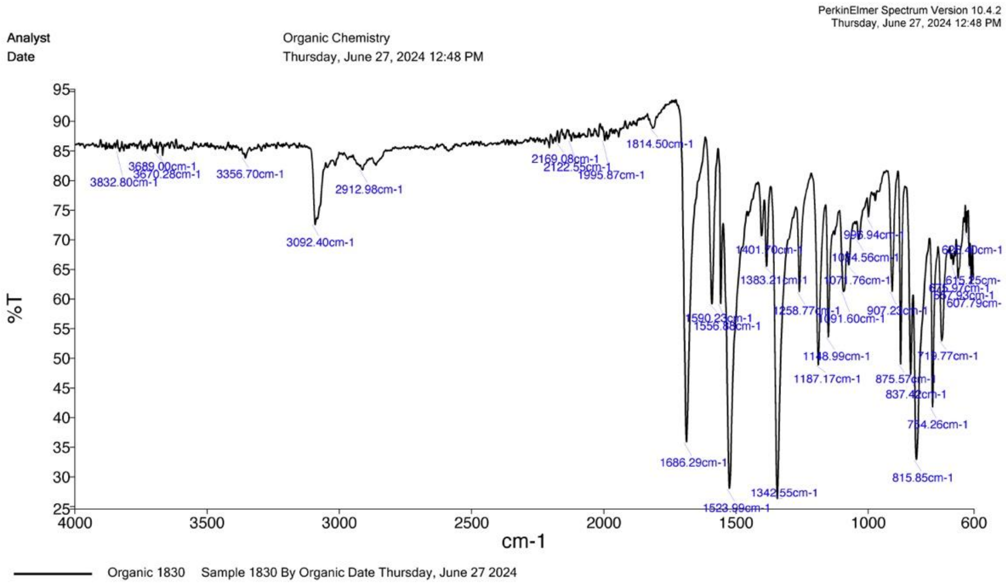

Figure S11: FT-IR spectrum of 4-bromo-2-nitrobenzaldehyde (**4**).

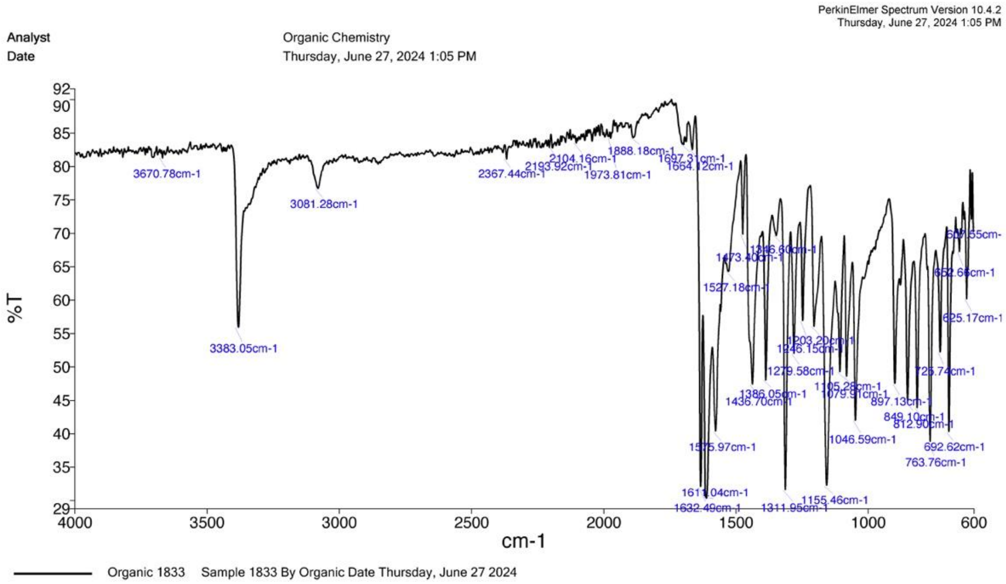

Figure S12: IR spectrum of 6,6’-dibromoindigo (**1**).

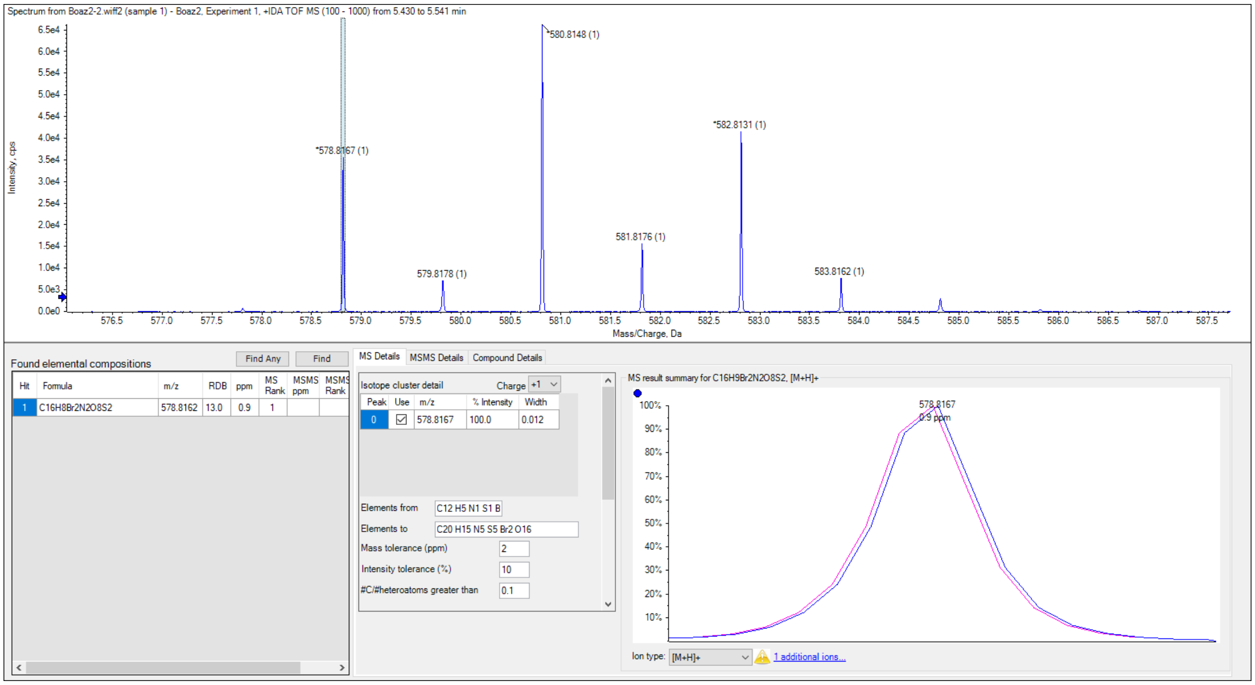

Figure S13: HRESIMS of 6,6’-dibromo-5,5’-indigodisulfonic acid disodium salt (**9**).

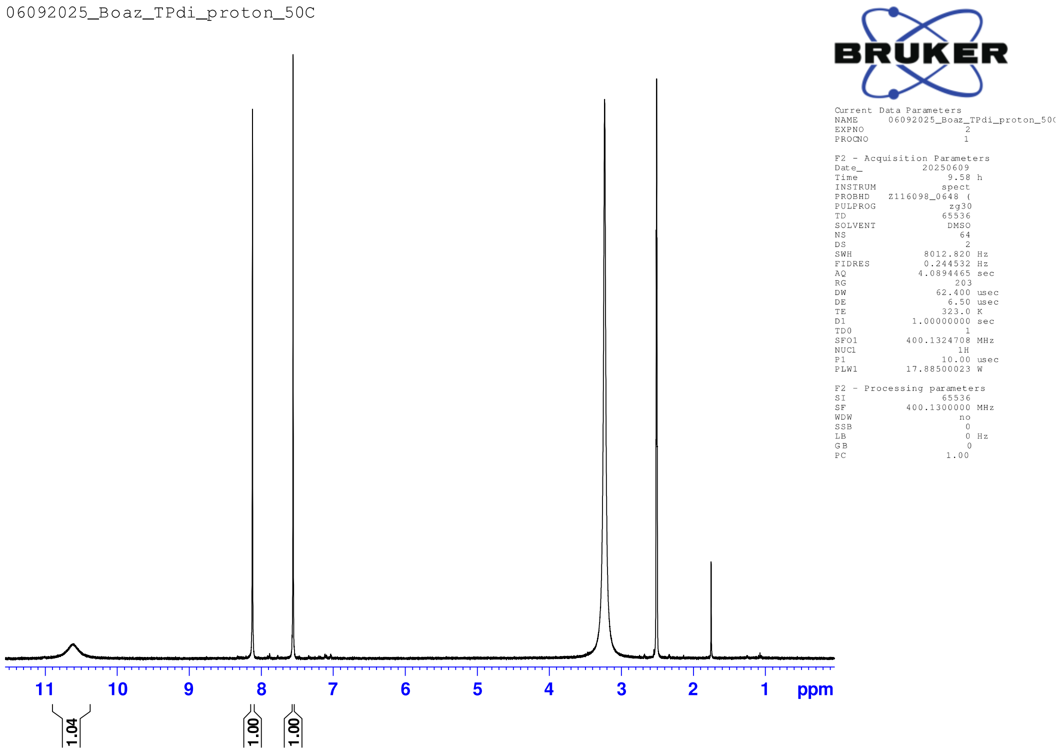

Figure S14: ^{1}H NMR (d_6DMSO, 50 °C) of 6,6’-dibromo-5,5’-indigodisulfonic acid disodium salt (**9**). The relatively poor solubility of this compound necessitated the use of elevated temperature to generate a sufficiently concentrated sample for analysis.

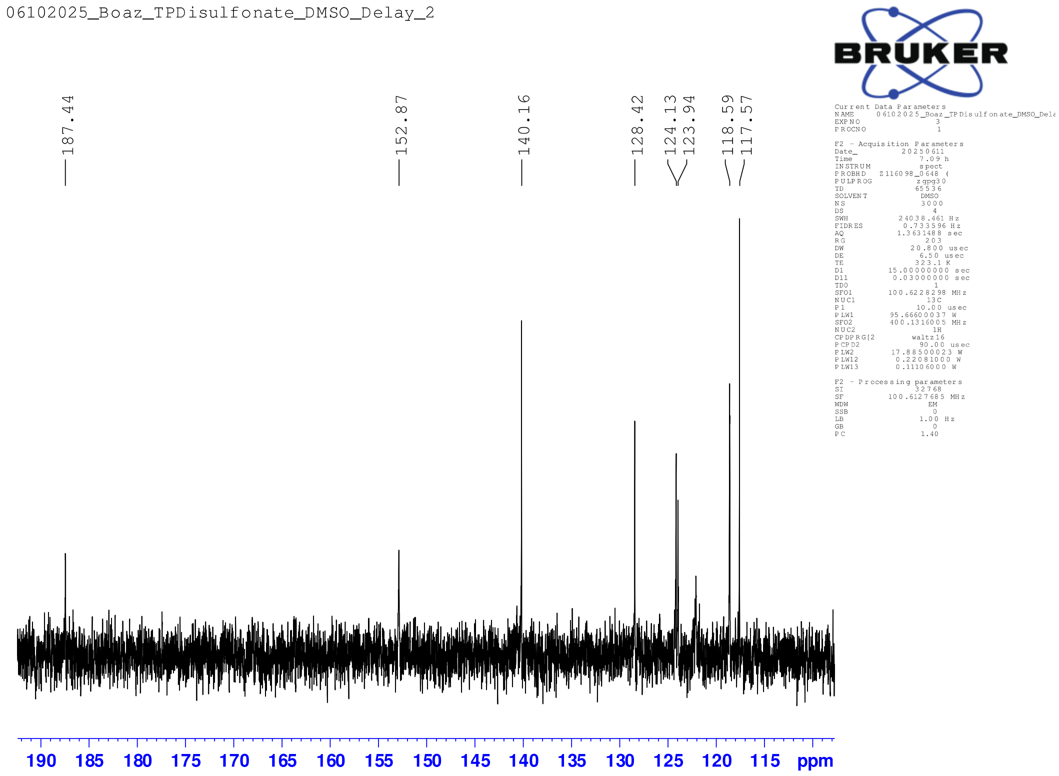

Figure S15: ^{13}C NMR (d_6DMSO, 50 °C) of 6,6’-dibromo-5,5’-indigodisulfonic acid disodium salt (**9**). The relatively poor solubility of this compound necessitated the use of elevated temperature to generate a sufficiently concentrated sample for analysis.

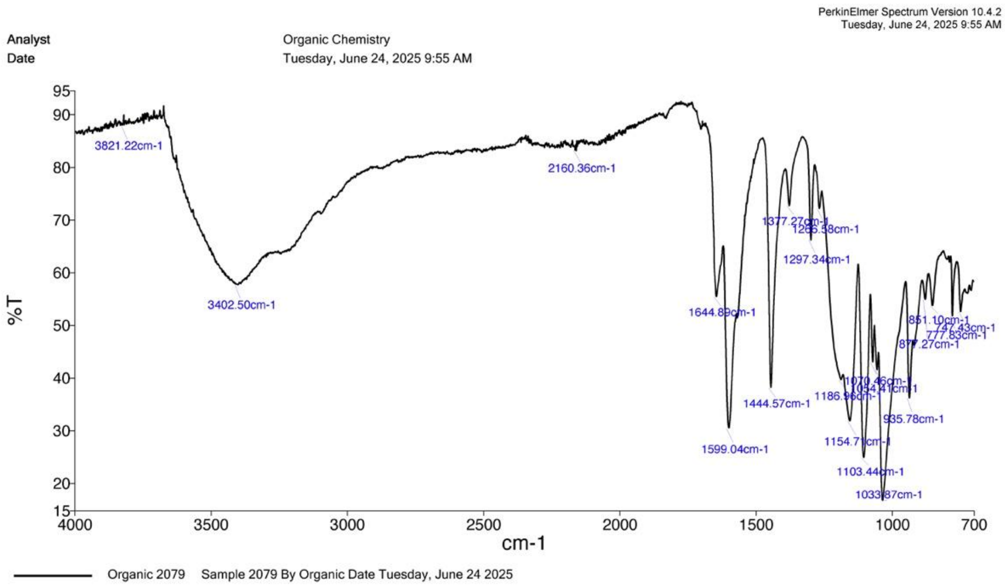

Figure S16: FTIR of 6,6'-dibromo-5,5'-indigodisulfonic acid disodium salt (**9**).

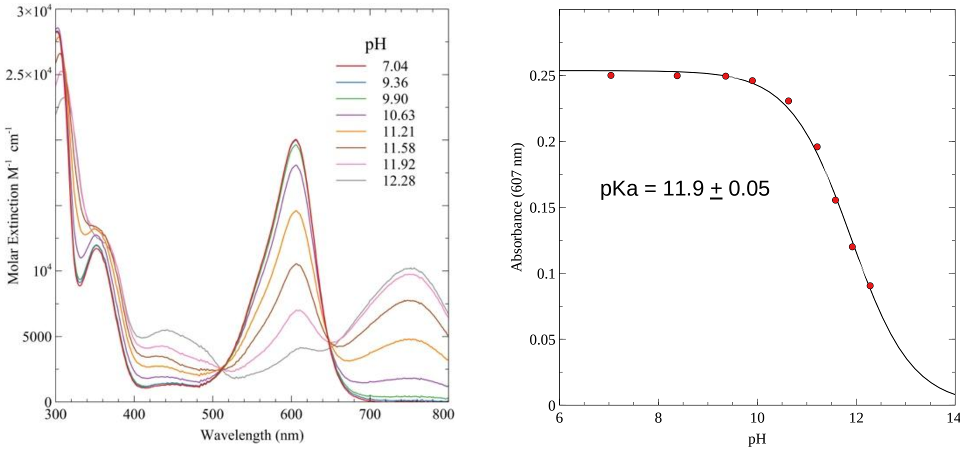

Figure S17: Left) Spectrophotometric titration of 6,6'-dibromo-5,5'-indigodisulfonic acid (**9**), in 100 mM phosphate/acetate buffer. Right) A plot of absorbance at 607 nm (10 mM 6,6'-dibromo-5,5'-indigodisulfonic acid) versus pH, yielding a pK_a of 11.9 $\pm$ 0.05.

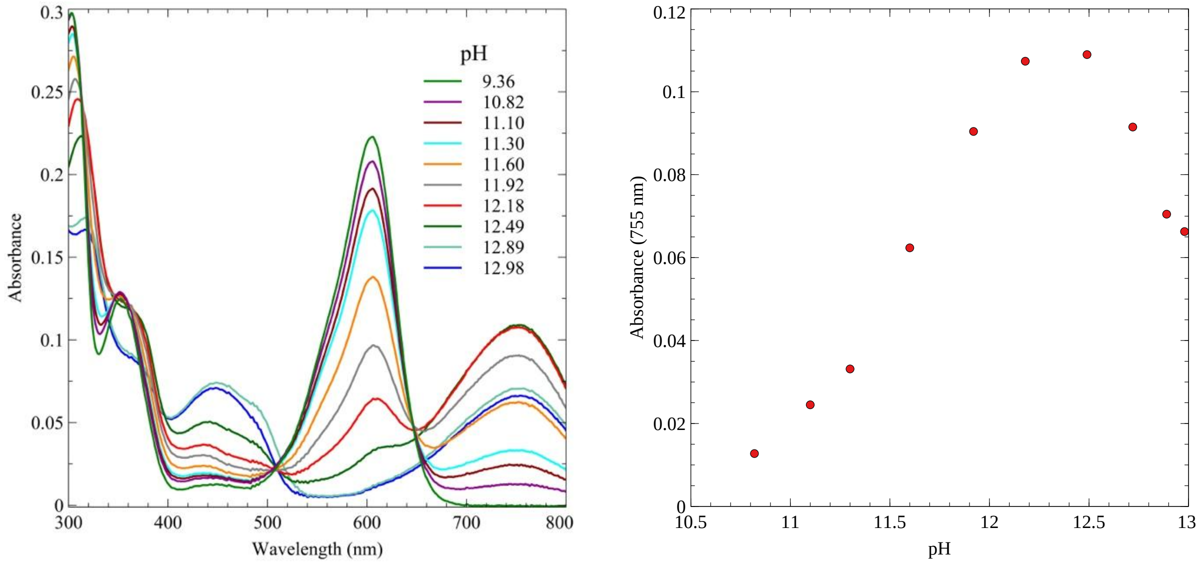

Figure S18: Left) Spectrophotometric titration of 6,6’-dibromo-5,5’-indigodisulfonic acid sodium salt (**9**), (10 mM) in unbuffered water. Please note that from a pH of 9.36 to 12.18, the change in spectra is isosbestic, indicating that there are only two species in solution. Above this pH, this is no longer the case, indicating the presence of at least one other species. It is suspected that a second pK_a exists above 12.5.

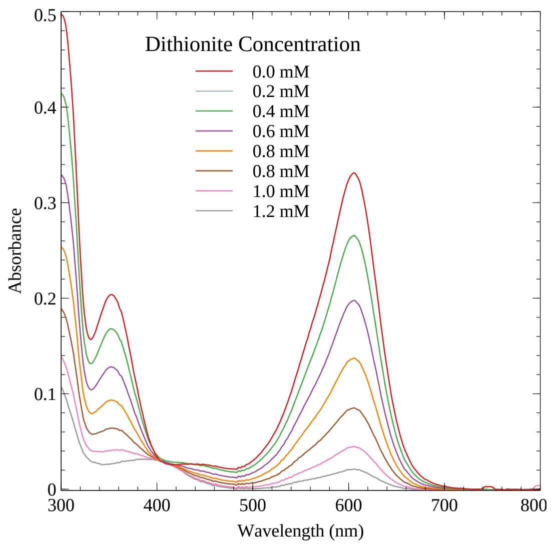

Figure S19: Reduction of 20 μM 6,6’-dibromo-5,5’-indigodisulfonic acid sodium salt (**9**), with sodium dithionite in 100 mM pH 7.00 phosphate/acetate buffer.

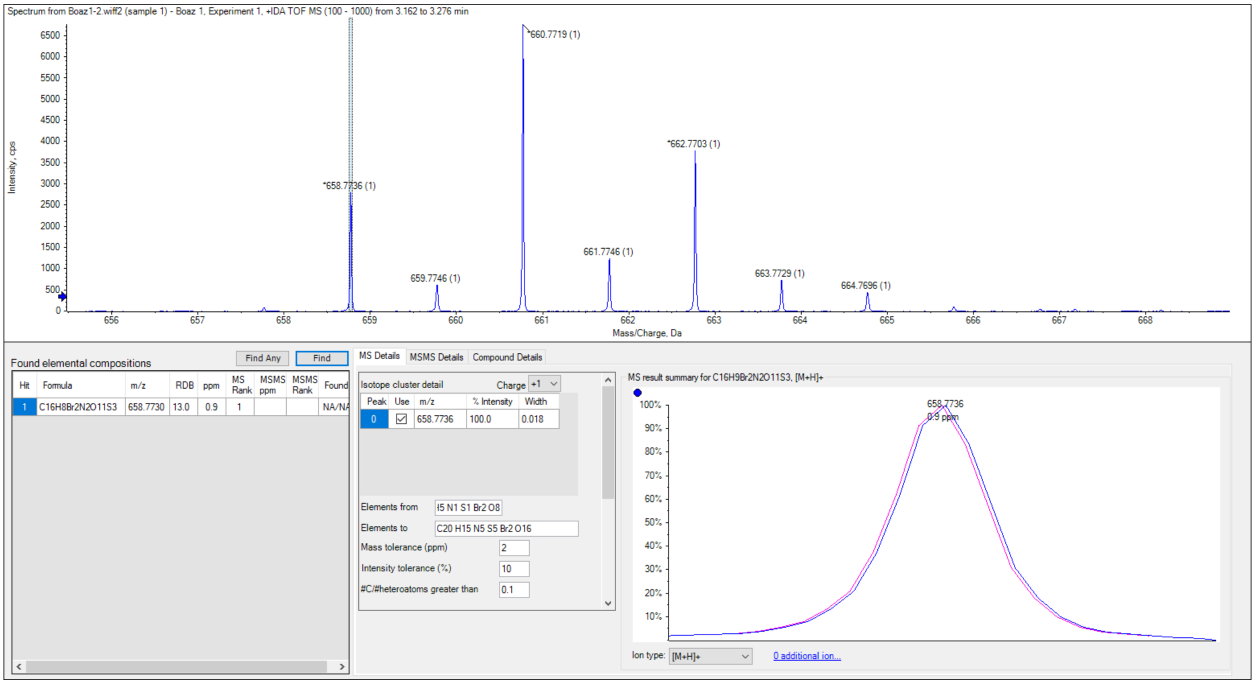

Figure S20: HRESIMS of 6,6’-dibromo-5,5’,7-indigotrisulfonic acid (**10**).

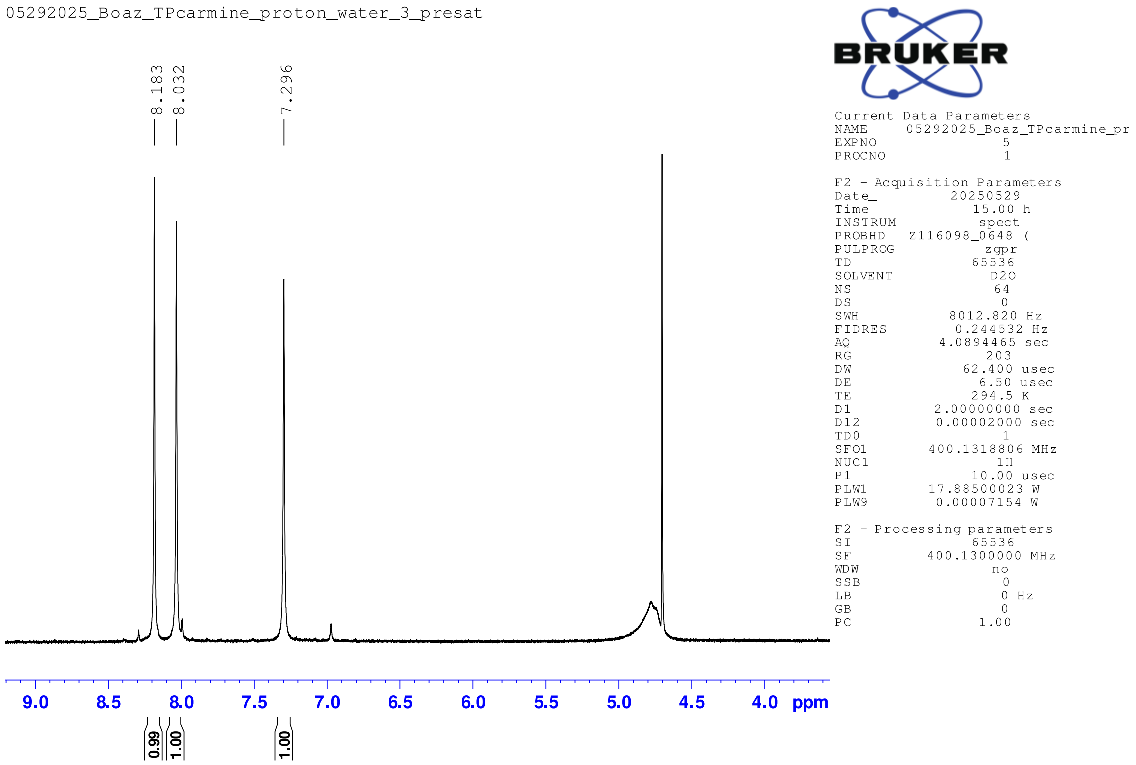

Figure S21: ^{1}H NMR (D_2O, 100 mM NaH_2PO_4, water suppression accomplished via presaturation) of 6,6'-dibromo-,5,5',7-indigotrisulfonic acid trisodium salt (**10**).

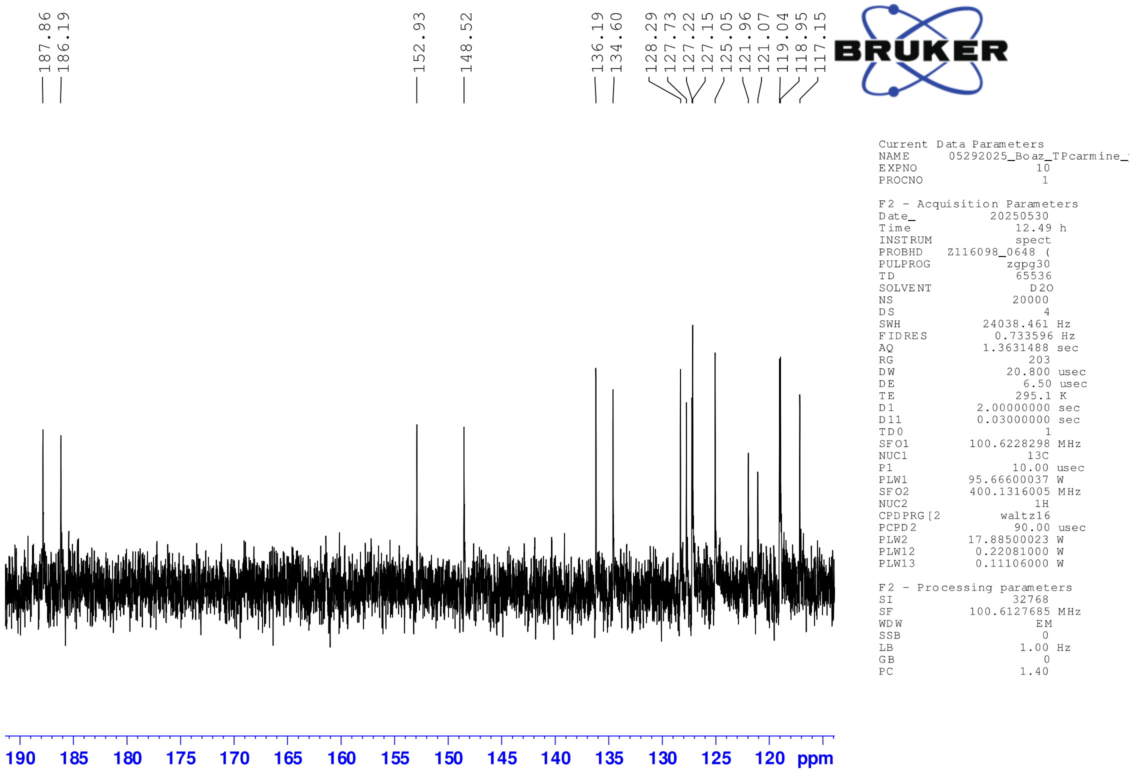

Figure S22: ^{13}C NMR (D_2O, 100 mM NaH_2PO_4) of 6,6'-dibromo-,5,5',7-indigotrisulfonic acid trisodium salt (**10**).

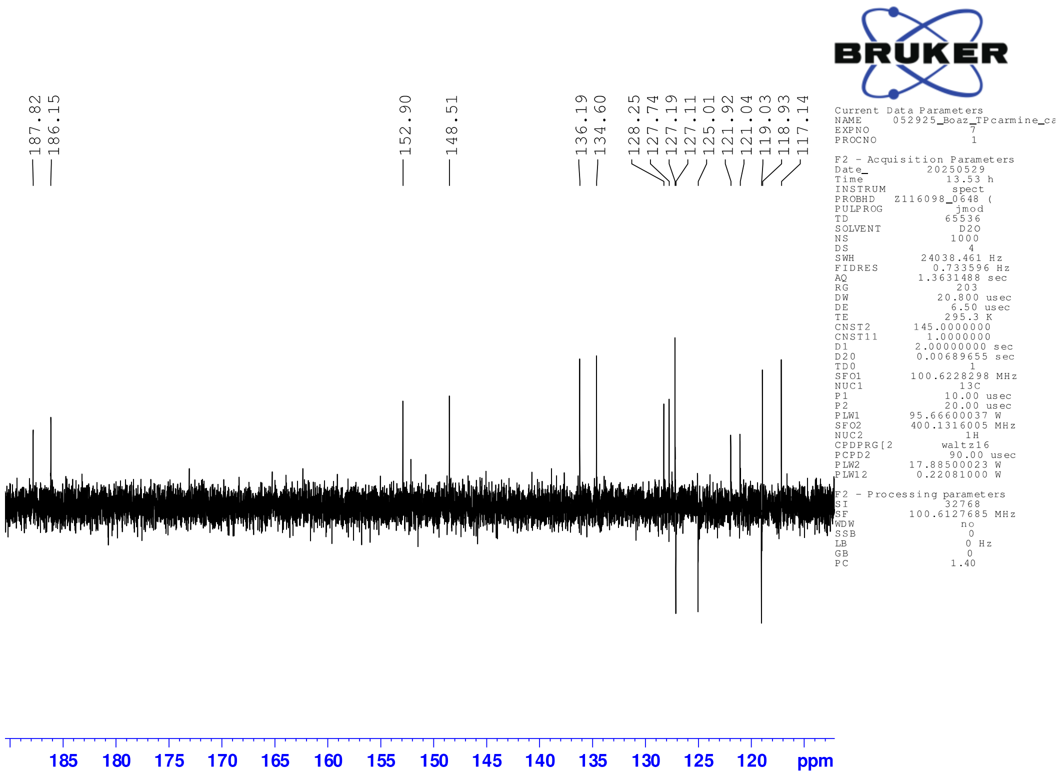

Figure S23: ^{13}C NMR (D_2O, 100 mM NaH_2PO_4, attached proton test) of 6,6'-dibromo-5,5',7-indigotrisulfonic acid trisodium salt (**10**).

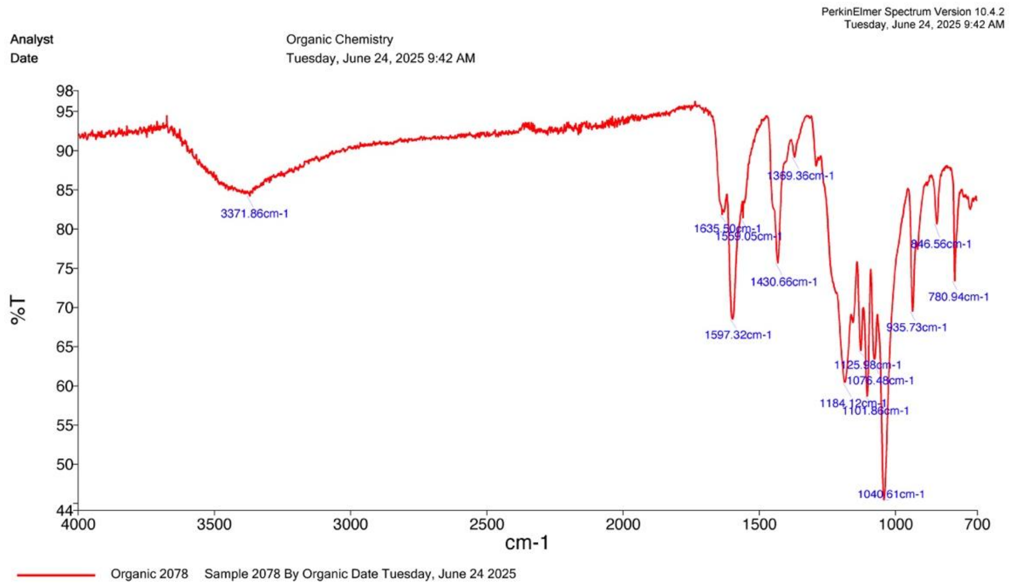

Figure S24. FT-IR of 6,6'-dibromo-5,5',7-indigotrisulfonic acid trisodium salt (**10**).

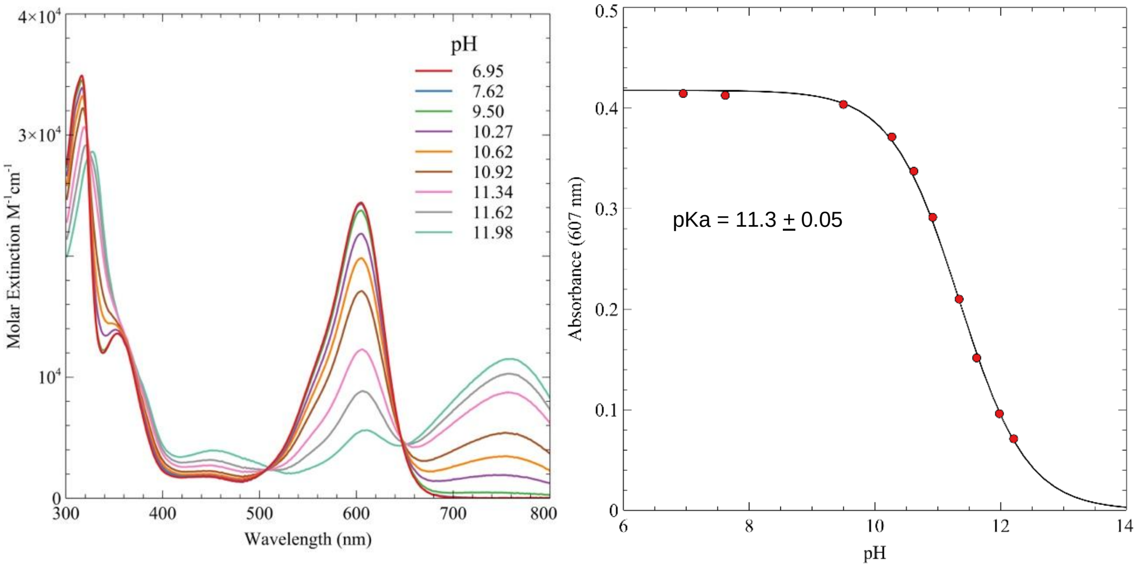

Figure S25: Left) Spectrophotometric titration of 6,6'-dibromo-5,5',7 indigotrisulfonic acid sodium salt (**10**), in 100 mM phosphate/acetate buffer. Right) A plot of absorbance at 607 nm versus pH in 100 mM phosphate acetate buffer, yielding a pK_a of 11.3 ± 0.05.

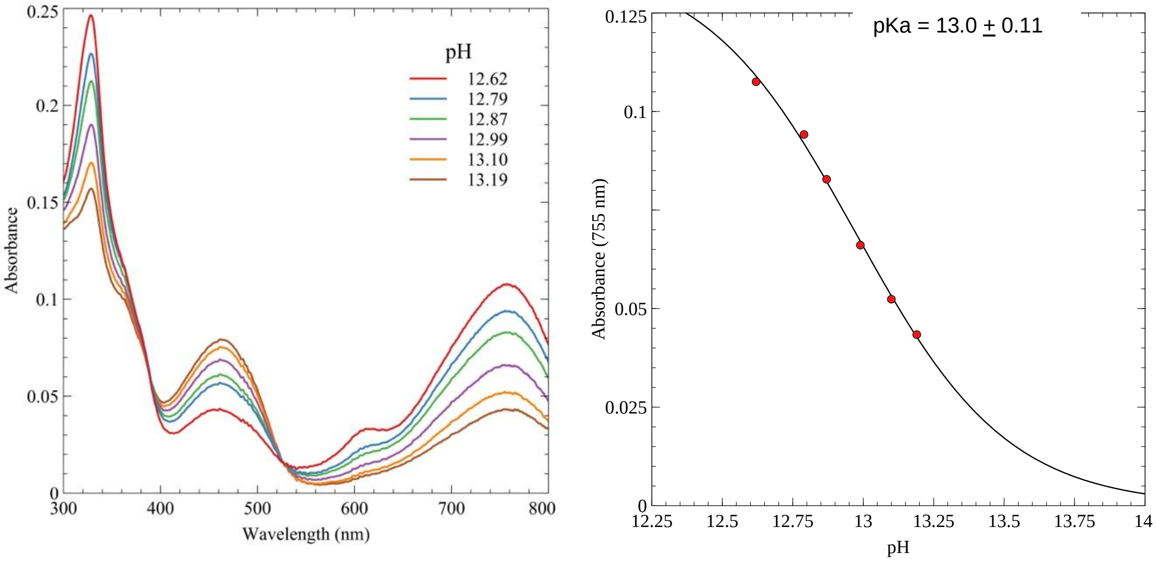

Figure S26: Left) Spectrophotometric titration of 6,6'-dibromo-5, 5',7-indigotrisulfonic acid trisodium salt (**10**), (10 mM) in unbuffered water from pH 12.62 to 13.19. Right) A plot of absorbance at 755 nm versus pH in unbuffered water indicates a pK_a of 13.0 ± 0.11.

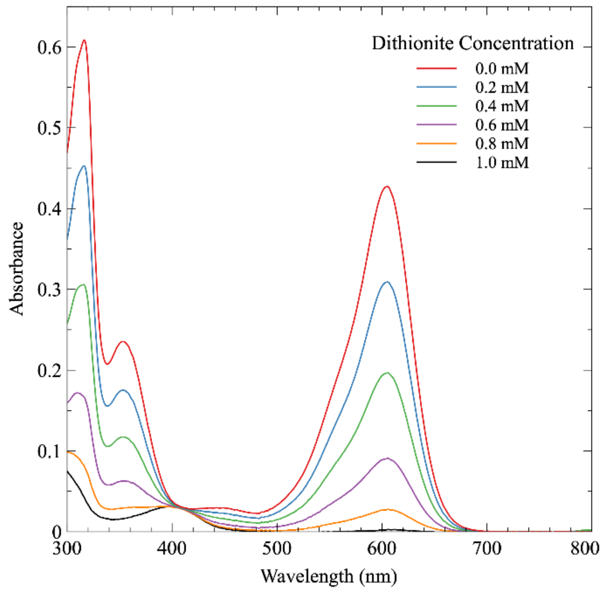

Figure S27: Reduction of 20 μM 6,6'-dibromo-5,5'-indigodisulfonic acid sodium salt (**9**), with sodium dithionite in 100 mM pH 7.00 phosphate/acetate buffer.

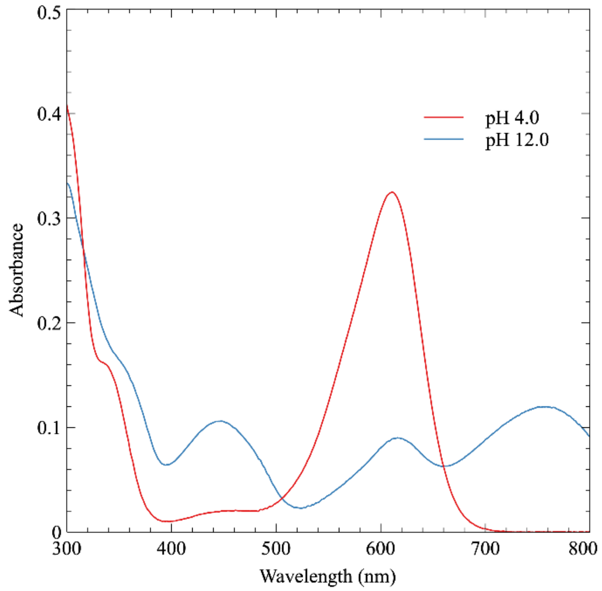

Figure S28. UV–vis spectrum of 20 μM indigo carmine in 100 mM phosphate/acetate buffer.

References

(1) Keinan, E.; Kumar, S.; Singh, S. P.; Ghirlando, R.; Wachtel, E. J. *Liquid Crystals* **1992**, *11*, 157–173. doi:10.1080/02678299208028981

(2) Conrad, W. E.; Rodriguez, K. X.; Nguyen, H. H.; Fettinger, J. C.; Haddadin, M. J.; Kurth, M. J. *Org. Lett.* **2012**, *14*, 3870–3873. doi:10.1021/ol3015804

(3) Rajesh, K.; Somasundaram, M.; Saiganesh, R.; Balasubramanian, K. K. *J. Org. Chem.* **2007**, *72*, 5867–5869. doi:10.1021/jo070477u

(4) Winum, J.-Y.; Bernaud, L.; Filhol, J.-S. *J. Chem. Educ.* **2021**, *98*, 1389–1396. doi:10.1021/acs.jchemed.0c01306

(5) Imming, P.; Imhof, I.; Zentgraf, M. *Synthetic Communications* **2001**, *31*, 3721–3727. doi:10.1081/SCC-100107023

(6) Sullivan, M. X.; Cohen, B.; Clark, W. M. *Public Health Reports (1896-1970)* **1923**, *38*, 1669. doi:10.2307/4576820
